# Breast cancer patient-derived whole-tumor cell culture model for efficient drug profiling and treatment response prediction

**DOI:** 10.1073/pnas.2209856120

**Published:** 2022-12-27

**Authors:** Xinsong Chen, Emmanouil G. Sifakis, Stephanie Robertson, Shi Yong Neo, Seong-Hwan Jun, Le Tong, Apple Tay Hui Min, John Lövrot, Roxanna Hellgren, Sara Margolin, Jonas Bergh, Theodoros Foukakis, Jens Lagergren, Andreas Lundqvist, Ran Ma, Johan Hartman

**Affiliations:** ^a^Department of Oncology-Pathology, Karolinska Institutet, Stockholm 17164, Sweden; ^b^Department of Clinical Pathology and Cancer Diagnostics, Karolinska University Hospital, Stockholm 17176, Sweden; ^c^Department of Computational Biology, Royal Institute of Technology, Science for Life Laboratory, Stockholm 17165, Sweden; ^d^School of Biological Sciences, Nanyang Technological University, Singapore 637551; ^e^Department of Breast Imaging, Södersjukhuset, Stockholm 11828, Sweden; ^f^Department of Clinical Science and Education, Södersjukhuset, Karolinska Institutet, Stockholm 11883, Sweden; ^g^Breast Center, Theme Cancer, Karolinska University Hospital, Stockholm 17176, Sweden

**Keywords:** breast cancer, whole-tumor cell culture, precision oncology, ex vivo culture, drug profiling

## Abstract

Breast cancer (BC) is a complex disease comprising multiple distinct subtypes with different genetic features and pathological characteristics. Although a large number of antineoplastic compounds have been approved for clinical use, patient-to-patient variability in drug response is frequently observed, highlighting the need for efficient treatment prediction for individualized therapy. Several patient-derived models have been established lately for the prediction of drug response. However, each of these models has its limitations that impede their clinical application. Here, we report that the whole-tumor cell culture (WTC) ex vivo model could be stably established from all breast tumors with a high success rate (98 out of 116), and it could reassemble the parental tumors with the endogenous microenvironment. We observed strong clinical associations and predictive values from the investigation of a broad range of BC therapies with WTCs derived from a patient cohort. The accuracy was further supported by the correlation between WTC-based test results and patients’ clinical responses in a separate validation study, where the neoadjuvant treatment regimens of 15 BC patients were mimicked. Collectively, the WTC model allows us to accomplish personalized drug testing within 10 d, even for small-sized tumors, highlighting its potential for individualized BC therapy. Furthermore, coupled with genomic and transcriptomic analyses, WTC-based testing can also help to stratify specific patient groups for assignment into appropriate clinical trials, as well as validate potential biomarkers during drug development.

## Significances

There is an urgent demand for discovering more accurate and predictive biomarkers and tools to facilitate precision oncology. Next-generation sequencing (NGS) technologies receive much attention but will probably never decipher the biological behavior of cancer cells completely. However, our established whole-tumor cell culture model, in combination with NGS tools and functional assays, could provide us with a platform to efficiently identify drug sensitivity and resistance for individual patients. We consider it a breakthrough over other existing ex vivo models by considering the tumor-stromal interactions to represent a more unbiased snapshot of the original patient's disease.

Breast cancer (BC) is the most common malignancy for women worldwide (1). In recent decades, significant improvements in BC screening and extensive use of adjuvant therapy have led to a current 5-y survival rate of around 90% (1). However, the current treatment strategies still largely rely on routine histopathological biomarkers: estrogen receptor (ER), progesterone receptor (PR), Ki67, and human epidermal growth factor receptor 2 (HER2) (2). ER expression confers strong indications for the response to endocrine therapy (3), whereas patients with HER2 overexpressing tumors can be effectively treated with anti-HER2 therapy (4, 5). However, such management of BC patients is insufficient to distinguish specific response patterns, which may cause a failure in therapy or overtreatment with unwanted side effects (6). Until recently, BCs have still been classified according to morphological growth patterns by microscopy (7). Nevertheless, morphological subtyping has a poor correlation with patient outcomes. Thus, it is still challenging to predict the effectiveness of a given treatment and which patient will most likely develop incurable metastatic disease. With the developments of gene expression technologies during the last two decades, a more precise subtyping tool with a stronger correlation to outcome and treatment response has been made possible (4, 8). Genomic sequencing also has the potential to identify patients carrying certain mutations with therapy predictive value, though only a few known somatic alterations are directly linked to treatment resistance (9, 10).

For decades, preclinical BC research has been conducted with dozens of cell lines as the in vitro manifestations of a heterogeneous disease. While cell lines are more feasible to access and handle experimental-wise, they do not fully represent the BC spectrum and provide minimal clinical relevance for individual patients (11). Patient-derived BC xenografts (PDX) can recapitulate the genotypes and phenotypes of the primary tumors to a certain extent and have been suggested as a candidate model for cancer therapy prediction. However, the high cost, engraftment variability, and low tumorigenicity during the establishment of BC PDX make it almost impossible to serve as a patient-specific model in reality (12). Hence, establishing advanced ex vivo models to guide precise oncology treatment is gaining attention. Earlier studies failed to set up primary BC cell cultures that could genuinely reflect their clinical phenotypes (13, 14), mainly due to the loss of ER expression in cells and failure to respond to endocrine therapies. A recent study by Sachs et al. has highlighted the importance of epithelium organoid culture for BC research and drug development (15). However, understanding the tumor–stroma cell interactions within the native tumor microenvironment (TME) is also of utmost importance in the pathogenesis and progression of cancer disease. Rising evidence supports the role played by tumor-associated stromal and immune cells in the acquisition of resistance to therapy (16, 17). Besides, it is time-consuming to generate adequate numbers of patient-derived organoids for drug profiling to give therapeutic recommendations. Therefore, by establishing whole-tumor cell culture (WTC) as an ex vivo model that could mimic the cell-to-cell contacts within the original TME, and efficiently investigating its behavior in response to a broad range of treatment regimens, we aim to create a more clinically relevant approach for BC drug profiling.

## Results

### Establishment and Characterization of BC WTCs.

**Fig. 1.**
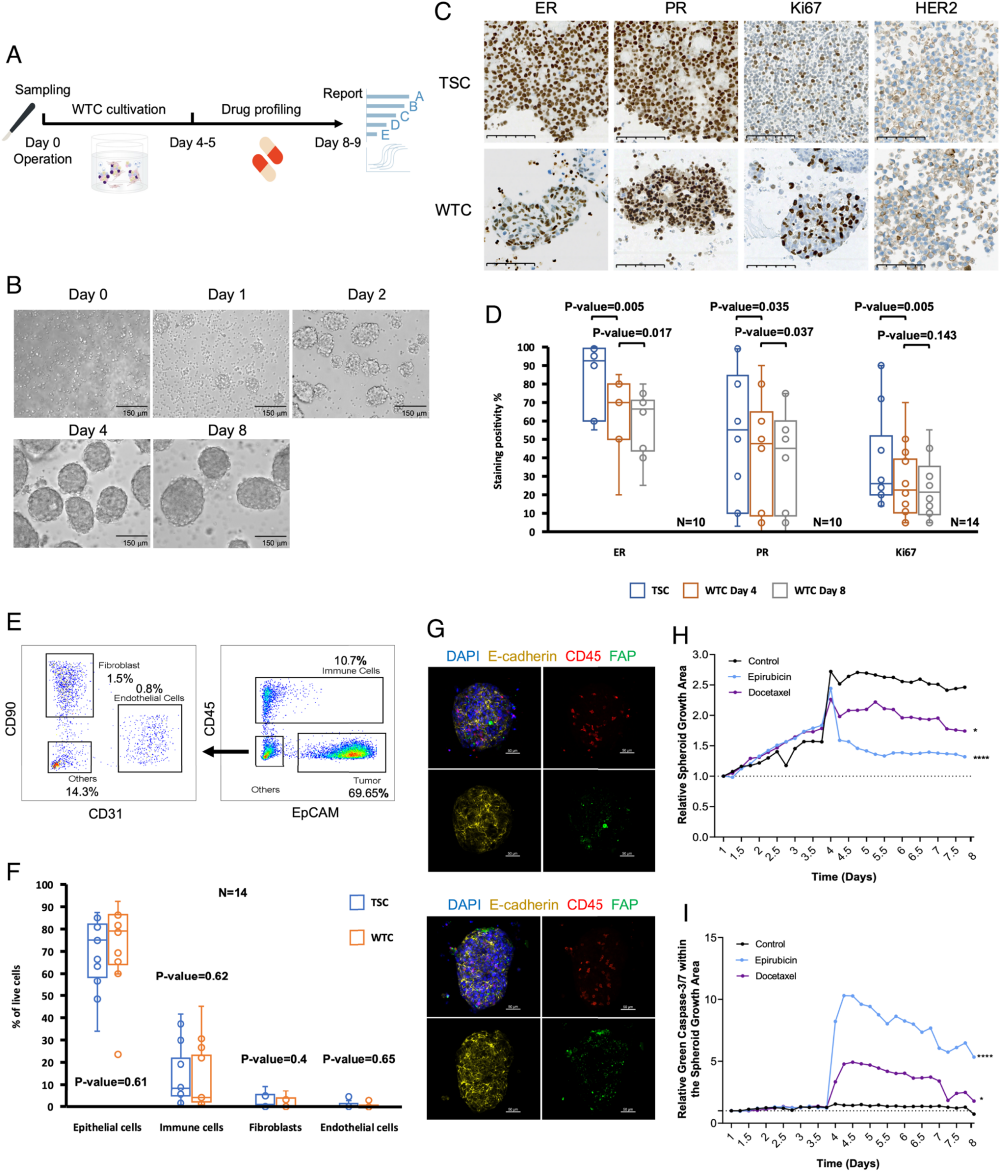
Biomarkers and cell composition in BC WTCs. (*A*) An overview of the WTC generation and drug profiling timeline. (*B*) Bright-field images show the time course of WTC spheroids formation during the culture period. (Scale bar, 150 μm.) (*C*) Immunohistochemical images of TSCs from parental tumors and subsequent WTCs culture. ER, PR, Ki67, and HER2 status are maintained in their established WTCs. (Scale bar, 100 μm.) (*D*) Box-and-whisker plots showing percentages of cell positivity of ER (N = 10), PR (N = 10), and Ki67 (N = 14) within TSC and WTC pairs. *P*-values were calculated using the Wilcoxon–Mann–Whitney test. (*E*) Representative flow cytometry analysis chart of cell compositions within paired TSCs and WTCs. (*F*) Bar plot showing the consistent fraction of cancer cells and various stromal cell populations within paired TSCs and WTCs (N = 14). *P*-values were calculated using the Wilcoxon–Mann–Whitney test. (*G*) Immunofluorescent co-staining of WTCs for epithelial cell marker E-cadherin (yellow), immune cell marker CD45 (red), and fibroblast cell marker FAP (green). Nuclei were counterstained (blue, Hoechst). (Scale bars, 50 μm.) (*H*) Relative area of spheroid growth (N = 3) and (*I*) relative green caspase 3/7 signal intensities within the spheroid area among WTC untreated control and chemotherapeutics treatment groups normalized to the day 1 reading. One-way ANOVA statistical analysis with Dunnett’s multiple comparisons to untreated control was performed with *P < 0.05, **P < 0.01, ***P < 0.001, and ****P < 0.0001.

The tumor scraping cells (TSCs) were obtained from primary BC patients who underwent either breast-conserving surgery or mastectomy and were immediately subjected to non-selective ex vivo culturing as described in the *Materials and Methods*. The cultured WTCs were ready for drug profiling after a 4 to 5-d cultivation period with a high success rate from all major tumor subtypes (*SI Appendix*, Fig. S1 *A*–*C*), and the individual drug response report could be generated within 10 d from TSC sampling (Fig. 1 *A* and *B*).

A representative BC model should be able to maintain clinically relevant biomarkers. Therefore, the expressions of ER, PR, Ki67, and HER2 in the TSC–WTC pairs were analyzed and confirmed by immunohistochemistry (IHC) (Fig. 1*C*). Hormone receptors are known to have predictive value for response to endocrine therapies, while HER2 status directly indicates the applicability of anti-HER2 therapies. We found that the expression of these receptors was indeed retained during the cultivation period, although the expression level was slightly decreased for PR and Ki67, and moderately decreased for ER (Fig. 1*D*). Generally, our WTC model resembled the primary pathological features and subtypes of the individual parental tumors.

Tumor–stromal cell interactions have long been recognized as important mechanisms in the pathogenesis and progression of cancer diseases (18). Recent studies also revealed that TME could play a key role in BC treatment resistance (17). Consequently, we aimed to establish a culture model that could maintain various stromal cell populations within the original patient TME for a more accurate prediction of drug responses. By performing flow cytometry analysis (FACS), we identified the various cell populations within the individual TSCs and WTCs, which were well retained during the cultivation period. Cancer cells were the majority, followed by various immune cells, fibroblasts, and a small population of endothelial cells (Fig. 1 *E* and *F* and *SI Appendix*, Fig. S1*D*). The localization of different cell types within WTCs was also shown by immunofluorescence staining and confocal microscopy analysis (Fig. 1*G*). In addition, we monitored the WTC spheroid formation and the apoptosis condition within the cultures by real-time imaging assays in both chemotherapy-treated and vehicle groups over time (*SI Appendix*, Fig. S1*E*). Upon exposure to epirubicin and docetaxel at 100 nM from day 4, the growth of WTC spheroids was significantly reduced compared with the untreated control (Fig. 1*H*). To confirm the growth inhibition we observed is treatment-specific, the apoptosis levels were also measured in parallel using a caspase-3/7 green labeling reagent in both conditions. Significantly increased apoptosis levels were only detected in the chemotherapy-treated spheroids, indicating the WTC has a stable culture condition that can truly reflect the efficacy of given treatments (Fig. 1*I*).

### WTCs Recapitulate the Genomic Landscape of the Original Tumors.

**Fig. 2.**
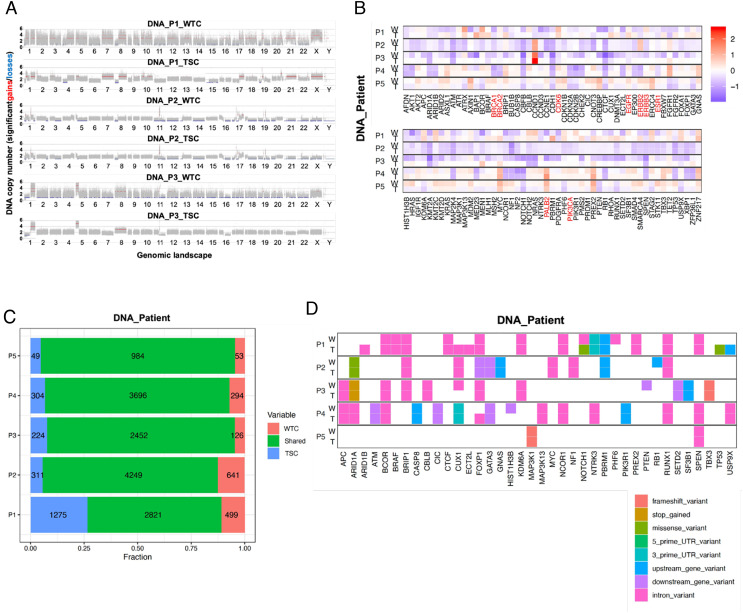
Genomic landscape of BC WTCs. Tumor-specific CNA patterns are retained by WTCs and display slightly higher signal amplitude than TSCs in both (*A*) genome-wide, and (*B*) BC-specific driver genes, where actionable genes for each patient are labeled with red text. Data are presented as BC TSCs (T) and WTCs (W) pairs (red: DNA copy number gains; blue: DNA copy number losses). In (*B*) CNAs are plotted as log2 ratios between the tumor and matched normal samples at the gene level for each patient. (*C*) The fraction of unique and shared short mutations (somatic SNVs and Indels) between WTCs and paired TSCs for each patient (red, green, and blue indicate the fraction of mutations unique to the WTCs, shared and unique to the TSCs, respectively). (*D*) Somatic mutations found in BC-specific driver genes and actionable genes are well conserved between the WTC (W) and TSC (T) pairs for each patient. The most deleterious variant (based on SnpEff) is presented per gene.

To evaluate the consistency of genomic spectra in WTCs compared with the parental tumors, we performed whole-genome sequencing (WGS) to characterize the copy number alterations (CNAs) and somatic mutation profiles in five random patient DNA sample pairs. After mapping the genetic distances among all samples, we observed that patient-specific sample pairs clustered together (*SI Appendix*, Fig. S2*A*). Since CNAs contribute to genetic variations and BC susceptibility, we compared genome-wide CNAs between original tumors and corresponding WTCs. Not surprisingly, DNA copy number gains and losses were highly similar within the patient sample pairs throughout the genome (Fig. 2*A* and *SI Appendix*, Fig. S2*B*). Furthermore, we focused on the BC-specific driver genes (19, 20) and actionable genes (21) to explore their respective CNA profiles (Fig. 2*B*). Again, the CNA patterns for these selected cancer genes were well recapitulated by WTCs and displayed a slightly higher signal amplitude than the original TSCs, which is in line with the genome-wide CNA pattern analysis (Fig. 2*A* and *SI Appendix*, Fig. S2*B*).

Somatic mutations are believed to be the driving force of cancer initiation and progression through the alteration of multiple cellular regulatory pathways (22). Besides, next-generation sequencing (NGS) techniques enable the identification of specific somatic alterations that can be efficiently targeted by certain drugs, thus improving patient outcomes (23). We assessed the somatic mutation profiles within the patient sample pairs to evaluate whether the WTCs can maintain these genomic features and their potential in clinical diagnosis. We first investigated the extent of shared alterations between WTC and paired TSC samples. On average, we found 79% of them to be identical (ranging from 61 to 91% between individual patients, Fig. 2*C*). Notably, most somatic mutations detected by single nucleotide variant (SNV) analysis in BC driver and actionable genes were very well conserved between the WTCs and original tumors, including the predicted loss-of-function mutations of *ARID1A, MAP3K1*, and *PIK3CA* (Fig. 2*D*). Moreover, the variant allele frequency (VAF) of all the detected SNVs were highly concordant between the TSC–WTC pairs with Pearson Correlation values all above 0.7, which further supported the maintenance of mutation patterns of the parental tumors by WTCs (*SI Appendix*, Fig. S2*C*). In summary, our WTC model could reliably recapitulate the parental BC tumor on the genomic level.

### Transcriptomic Analysis of BC WTCs.

**Fig. 3.**
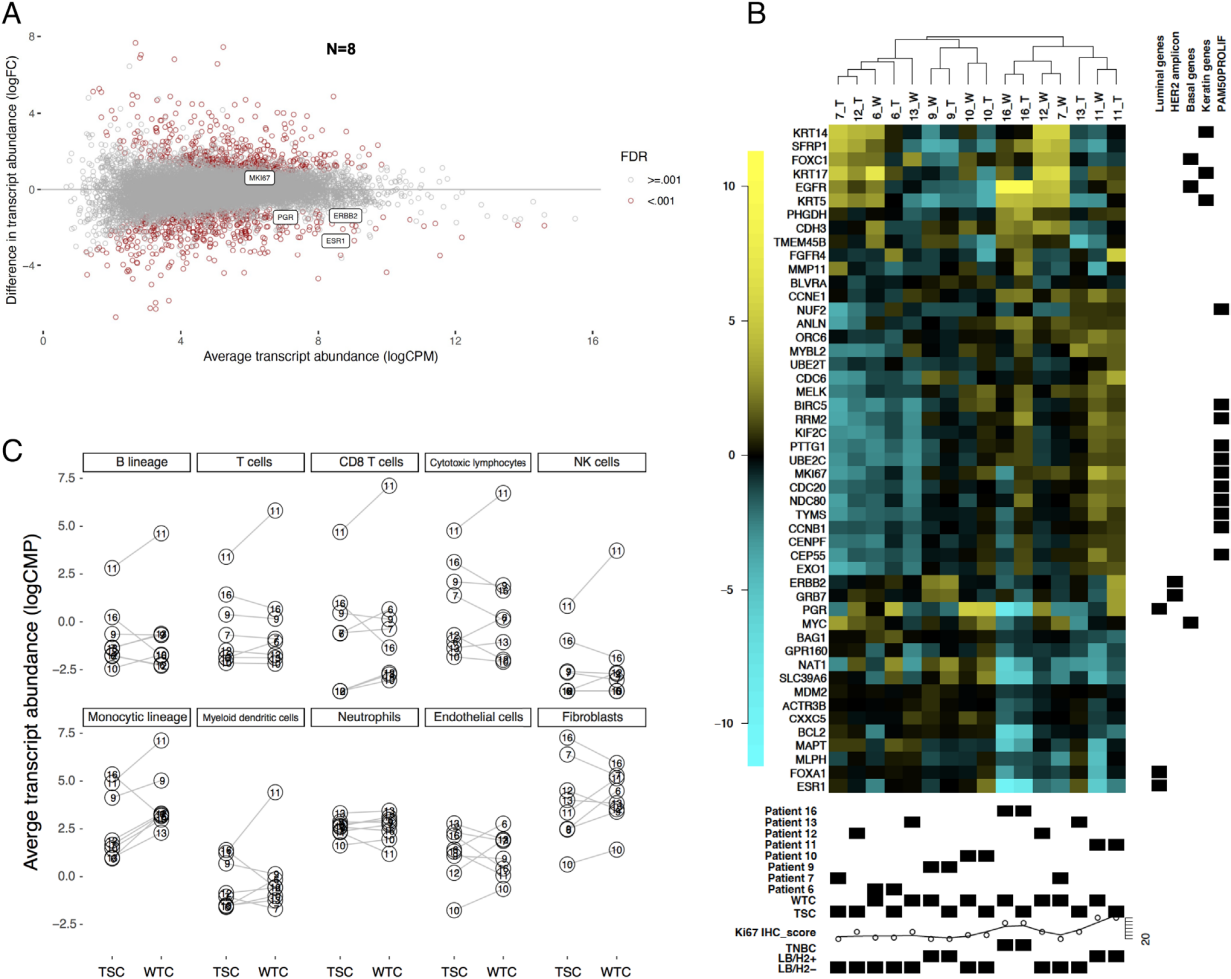
Gene expression analysis of BC WTCs by RNA sequencing. (*A*) Changes in transcript abundances between BC WTC and TSC conditions within patients (N = 8) are presented in a mean-difference plot. Transcripts with FDR < 0.001 are indicated in red color. *ESR1PGRERBB2*, and *MKI67* are also marked. logFC: log2-fold change; logCPM: average log2-counts per million; FDR: false discovery rate. (*B*) Clustering of paired TSC (T) and WTC (W) samples based on the PAM50 intrinsic gene-set. Specifically, average-linkage hierarchical clustering was performed with the one minus correlation metric, after gene-centering using the median of TSC samples from the surgical specimens. The heatmap cells are color-coded in comparison with the overall median gene expression of TSCs (yellow: higher than the overall median gene expression of TSCs; black: overall median of TSCs; blue: lower than the overall median of TSCs). Annotation rows show patient ID, sample type (TSC/WTC), Ki67 value, and clinical IHC-based surrogate subtype assignment of the surgical specimen. Annotation columns indicate the PAM50 genes category. PAM50PROLIF: PAM50 proliferation index. LB/H2−: luminal B-like HER2-negative; LB/H2+: luminal B-like HER2-positive; TNBC: triple-negative. (*C*) The absolute transcript abundance of eight immune and two stromal cell populations is estimated by the Microenvironment Cell Populations-counter (MCP-counter) method. Numbers in circles correspond to patient IDs. The thick gray line and shading represent a local polynomial regression smoother (LOESS) with its 95% confidence band, respectively.

Gene expression profiling is now well established to classify BC into intrinsic subtypes (24). Therefore, the expression of BC genes was assessed in a set of eight random pairs of original TSCs and WTCs by RNA sequencing. The data revealed that the transcript abundances of *PGR*, *MKI67,* and *ERBB2* were well maintained in the WTCs, in line with the findings on protein level. Though the *ESR1* transcript exhibited a significantly lower abundance in WTCs (FDR < 0.001; Fig. 3*A*), IHC data revealed that the ER protein level was still retained. Five out of eight pairs of TSC and WTC, consisting of two luminal B-like HER2-positive (LB/H2+), one triple-negative (TNBC), and two luminal B-like HER2-negative (LB/H2−) tumors, defined by IHC-based surrogate subtypes, showed a conserved PAM50 gene expression profile and clustered together (Fig. 3*B*). The other three cases that failed to cluster together were all LB/H2− cases and varying expression levels were mostly observed among the proliferation-related genes and *ESR1*. Additionally, by mapping the transcriptomic distances among all samples with global gene expressions, we observed that most of the WTCs stay close to the paired TSCs, and tumor samples with different subtypes were separated (*SI Appendix*, Fig. S3*A* and List S1). However, the sample-pair pattern could be affected within reason if the parental tumor samples were highly similar from the beginning, such as P6 and P12.

Both differential expression and gene-set enrichment analyses indicated a slightly more aggressive status in the WTCs compared with the paired TSCs (*SI Appendix*, Tables S1 and S2) with inflammatory response, TNF-a signaling, as well as PI3K-AKT-mTOR signaling among the most up-regulated pathways. The absolute transcript abundances of eight immune and two stromal cell populations were retained in all the TSCs-WTCs. In addition, comparable transcript levels were observed for most of the patient sample pairs. Interestingly, the TNBC patient RNA-16 and the LB/H2+ patient RNA-11 exhibited the highest and most variable abundances in immune cell populations, while all the HER2-negative luminal cases showed a more consistent pattern before and after culturing (Fig. 3*C*).

### Gene Expression Analysis of WTCs to Predict Endocrine Therapy Response.

**Fig. 4.**
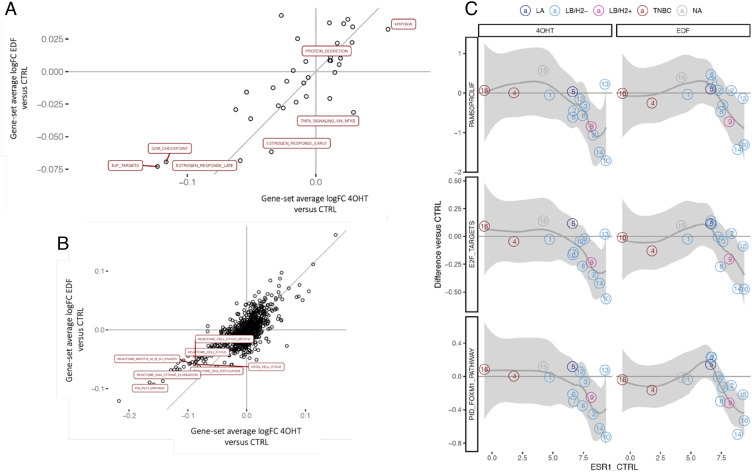
BC WTCs allow ex vivo evaluation of patient-specific sensitivity to endocrine therapy by RNA sequencing. Scatter plots of gene-set average logFC for the two treatment comparisons (4OHT versus untreated, and EDF versus untreated), using either (*A*) the Hallmarks, or (*B*) the Canonical pathways gene-set collection. The top five gene sets (ranked by the NES) that are statistically significant (FDR < 0.01) in any of the comparisons are marked with their corresponding names. logFC: log2-fold change; NES: normalized enrichment score; FDR: false discovery rate. (*C*) Change in proliferation capacity as a surrogate marker of endocrine treatment response in relation to *ESR1* transcript abundance. Numbers in circles correspond to patient IDs, color-coded based on the clinical IHC-based surrogate subtype assignment on surgical specimens. The thick gray line and shading represent a local polynomial regression smoother (LOESS) with its 95% confidence band, respectively. PAM50PROLIF: PAM50 proliferation index; E2F_TARGETS: MSigDB Hallmark gene-set including genes encoding cell-cycle related targets of E2F transcription factors; PID_FOXM1_PATHWAY: MSigDB Canonical/PID pathway of FOXM1 transcription factor network. LA: luminal A-like; LB/H2−: luminal B-like HER2-negative; LB/H2+: luminal B-like HER2-positive; TNBC: triple-negative; NA: not applicable.

4-hydroxytamoxifen (4OHT) and endoxifen (EDF) are the main metabolites of tamoxifen citrate and have been proposed to exert the same treatment benefit for ER-positive BC patients (25). Thus, we performed RNA sequencing of WTCs derived from 16 random patients, ex vivo treated with 4OHT and EDF separately for 96 h. We first evaluated the top 500 most varying transcripts triggered by both treatments. Not surprisingly, while the intrapatient heterogeneity was evident, the transcripts altered by both treatments still clustered together within the same patient, indicating that 4OHT and EDF exerted similar effects (*SI Appendix*, Fig. S3*B*). Again, this observation was further strengthened by the comparison of the log fold changes (treated versus untreated) of all transcripts induced by these two treatments, with a high agreement between 4OHT and EDF for the same patient (*SI Appendix*, Fig. S3*C*). We further studied *MKI67* transcript abundances, PAM50 proliferation index (26), and AURKA module (27) score as surrogate markers of treatment response in each patient’s WTC. As expected, the proliferation-related genes were down-regulated only among ER-positive patients in both treatment groups (*SI Appendix*, Fig. S3*D*). Hence, these observations support that WTCs could maintain functional ER status as their parental tumors. Furthermore, the gene-set enrichment analysis (GSEA) identified a high degree of agreement among the signaling pathways between the two treatment groups (using either the Hallmarks or the Canonical pathways gene-set collection), indicating that there is no significant difference in the molecular pathways induced by 4OHT and EDF treatment (Fig. 4 *A* and *B* and *SI Appendix*, Tables S3–S6).

To further investigate if our approach could be applied for the prediction of individual patient endocrine therapy response, we employed the *ESR1* transcript abundance as an example of a known predictor of tamoxifen treatment. Changes in the PAM50 proliferation index, the Hallmark E2F targets, and the FOXM1 pathway gene-sets were used as response predictive biomarkers. Interestingly, we identified a trend in the relation between the expected response to endocrine treatment and *ESR1* transcript abundance at baseline, apart from patient RNA-13, who presented an outlying behavior (Fig. 4*C*). In summary, proliferation-based gene expression analysis indicated that WTCs are responsive to tamoxifen metabolites. More importantly, our results suggest that it could be possible to distinguish tamoxifen-responding patients versus nonresponders by an ex vivo assay.

### Patient Sample Characterizations for Drug Profiling and Gene Expression Analysis.

Once the experimental reliability of the BC WTC method was confirmed, we evaluated its potential as an efficient and functional drug profiling platform. We randomly collected fresh tumor biopsies from 45 patients as a pilot cohort including 8 LA, 21 LB/H2−, 4 LB/H2+, 2 H2+ (HER2-positive nonluminal), and 10 TNBC tumors (*SI Appendix*, Fig. S4*A*). The average tumor size was 30 mm (range 3 to 125 mm; *SI Appendix*, Fig. S4*B*) with around 30% of the tumors less than 2 cm in diameter. We were able to establish WTCs from those tumors that are usually considered technically challenging to generate ex vivo cultures. We also acquired the corresponding routine formalin-fixed paraffin-embedded (FFPE) tissue samples (patients 1 and 41 were missing) and performed gene expression analysis and pathway scoring by the NanoString BC 360 (BC360) panel. Patient details are summarized in supplementary data (*SI Appendix*, List S1).

### Drug Profiling of Clinically Relevant Subgroups.

Next, we wanted to investigate individual patient tumor responses to routine therapies as well as experimental treatment options base on our WTC method. We selected a small set of drugs, which contained both standard chemotherapy and targeted therapy for BC, as well as a few drugs currently enrolled in phase II-III trials (*SI Appendix*, List S3). Then, we performed cell viability assays, and the drug sensitivity scores (DSSs) were calculated as described in the *Materials and Methods*. We also carried out unsupervised clustering analysis for both the compounds and the tumors based on their individual DSSs.

**Fig. 5.**
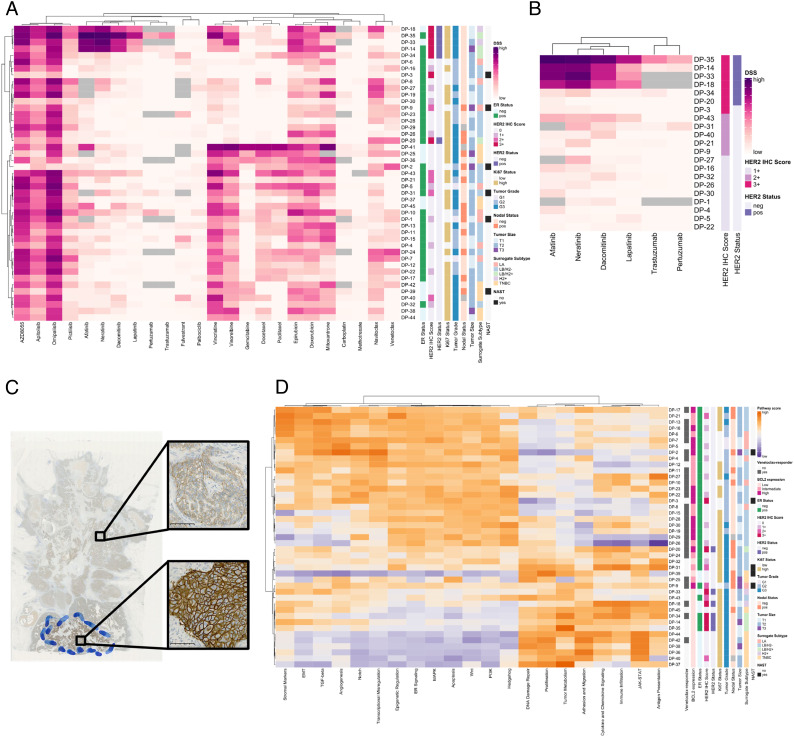
BC WTCs allow ex vivo drug screening. (*A*) Heatmap based on the DSSs from established WTCs. DSSs were clustered using Ward’s hierarchical clustering algorithm and Spearman’s rank-based correlation coefficient. Cells are color-coded by the DSS value (light gray color indicates not available data). Rows correspond to individual patients, and columns represent compounds. Samples are annotated with the clinical characteristics of the original breast tumors. (*B*) Heatmap based on the DSSs from established WTCs for drugs targeting the HER2 signaling pathway and only for the subset of patients with HER2 IHC score > 1. DSSs were clustered using Ward’s hierarchical clustering algorithm and Spearman’s rank-based correlation coefficient. Cells are color-coded by the DSS value (light gray color indicates not available data). Rows correspond to individual patients, and columns represent the compounds targeting the HER2 signaling pathway. Samples are sorted by decreasing the HER2 IHC score of the original breast tumors. (*C*) Intratumoral heterogeneity of HER2 IHC from different regions of the DP-20 tumor. (*D*) Heatmap based on the signaling pathway scores from FFPE tissue samples of WTC drug-profiled patients (NanoString BC360 panel derived data). Pathway scores were clustered using Ward’s hierarchical clustering algorithm and Spearman’s rank-based correlation coefficient. Cells are color-coded by the score values. Scores are displayed on the same scale via a Z-transformation. Rows correspond to individual patients, and columns represent pathways. Samples are annotated with venetoclax responsiveness and with the clinical characteristics of the original breast tumors. Venetoclax responsiveness was based on the DSSs from established WTCs, and a cutoff of one was used to separate the nonresponders (DSS < 1) from the responders (DSS ≥ 1). LA: luminal A-like; LB/H2−: luminal B-like HER2-negative; LB/H2+: luminal B-like HER2-positive; H2+: HER2-positive nonluminal; TNBC: triple-negative, IHC-based surrogate subtypes. NAST: neoadjuvant systemic treatment.

Several relevant drug clusters were identified from the analysis, led by the PI3K/mTOR and the EGFR/HER2 inhibitors. The microtubule/tubulin-associated drugs and the topoisomerase II inhibitors also clustered accordingly, followed by the BCL-2 inhibitors (Fig. 5*A*). As expected, drugs dependent on liver metabolism, including selective ER modulators, achieved no or only marginal DSSs (*SI Appendix*, Fig. S5*A*). Interestingly, when accessing the patient characteristics, we found that the ER-positive patients were identified as two major groups divided by a small TNBC cluster, while the HER2-positive patients were clustered together (Fig. 5*A*). Notably, the most distinguishable difference between the two ER-positive clusters was the response to taxanes (docetaxel and paclitaxel) and vinca alkaloid drugs (vincristine and vinorelbine).

Next, we focused on the HER2-expressing patients and could confirm that responses to specific inhibitors were in concordance with both HER2 protein expression and the clinically defined HER2 status (Fig. 5*B*). Our statistical analysis also suggested that the HER2 status acted as the only parameter to predict response to HER2-targeted drugs, in agreement with clinical guidelines (*SI Appendix*, Fig. S6 *A*–*F*). Of note, the WTC from patient drug profiling (DP)-20 showed minimal sensitivity to any of the ex vivo anti-HER2 treatments, though considered as a HER2-positive tumor (Fig. 5*B*). After reexamining the routine diagnostic material, we found out that it expressed HER2 in a heterogeneous pattern, with only 15% of cells scored IHC 3+ forming a hotspot area, while the other cells scored 1 to 2+, which might explain its unresponsiveness toward the targeted therapies (Fig. 5*C*). Another example is DP-3, though the resected tumor had a HER2 IHC score of three, the WTC established from it showed negligible sensitivity against any tested anti-HER2 drugs (Fig. 5*B*). When looking closer, we found that tumor DP-3 was considered HER2 negative with an IHC score of 1+ from examined core needle biopsy before the patient received neoadjuvant therapy as for a luminal B HER2-negative tumor. However, the HER2 status switched to an IHC score of 3+ during the treatment period, but SISH results determined it still as HER2-negative (*SI Appendix*, List S1). From Fig. 5*A*, we also can see that DP-20 and DP-3 were not clustered together with the other IHC-based HER2 3+ tumors on the top of the heatmap.

Venetoclax is a selective BCL-2 inhibitor and is currently investigated for the treatment of patients with ER and BCL-2 positive breast tumors. Interestingly, our results showed that venetoclax drug sensitivity significantly correlated with both *BCL2* transcript abundance and ER positivity (*SI Appendix*, Fig. S5 *B* and *C*). In addition, tumor grade, Ki67, and HER2 status implied their potential relevance for venetoclax treatment efficiency through negative correlations (*SI Appendix*, Fig. S6*H*). Besides, we explored the scores of biologically important pathways covered by the BC360 gene expression panel among the drug-profiled tumors. Here, we observed two distinct patient subgroups by unsupervised clustering, representing venetoclax responders and nonresponders (Fig. 5*D*). It is worth mentioning that the high scores of ER signaling, apoptosis, PI3K, Wnt, and MAPK pathways were hallmarks of the venetoclax responding patients, whereas the high scores of DNA damage response, tumor metabolism, JAK-STAT, immune response, and proliferation pathways highlighted the nonresponders. Notably, most of the responders expressed intermediate to high levels of *BCL2* and were ER-positive. In contrast, the majority of nonresponders expressed lower levels of *BCL2* and were mostly HER2-positive or ER-negative patients.

Palbociclib is a selective CDK4/6 inhibitor used to treat patients with ER-positive, HER2-negative metastatic disease but has also been studied as adjuvant therapy in several clinical trials (28, 29). In our analysis, we observed that only WTCs from ER-positive patients responded to palbociclib (*SI Appendix*, Fig. S5*D*). Gemcitabine is recommended for patients with advanced BC, and in our assay, it showed significantly higher DSSs among the WTCs derived from ER-negative tumors compared with ER-positive tumors (*SI Appendix*, Figs. S5*E* and S6*G*). Despite the fact that response differences between individual patient WTCs do exist, the three topoisomerase II inhibitors doxorubicin, epirubicin, and mitoxantrone showed robust treatment responses irrespectively of prognostic parameters including ER status, tumor sizes, nodal status, and Ki67 (*SI Appendix*, Fig. S6 *I*–*K*). This observation is largely in line with a meta-analysis that revealed tumor characteristics had minor effects on BC patient outcomes after anthracycline-based treatment (30, 31).

At last, we performed a drug profiling analysis on WTCs derived from different regions of a large TNBC tumor DP-45 to explore intratumoral heterogeneity in drug response (*SI Appendix*, Fig. S7*A*). Drugs with DSS > 0 in any of the tumor samples were plotted accordingly as a heatmap, where most of the therapies showed responses across the whole tumor area. However, drugs such as docetaxel and doxorubicin also showed sensitivity variations between different tumor regions (*SI Appendix*, Fig. S7*B*). Furthermore, several drugs, including carboplatin and gemcitabine, were found effective only in certain regions of the tumor.

### WTCs Accurately Predict Patient Response to Neoadjuvant Treatments.

**Fig. 6.**
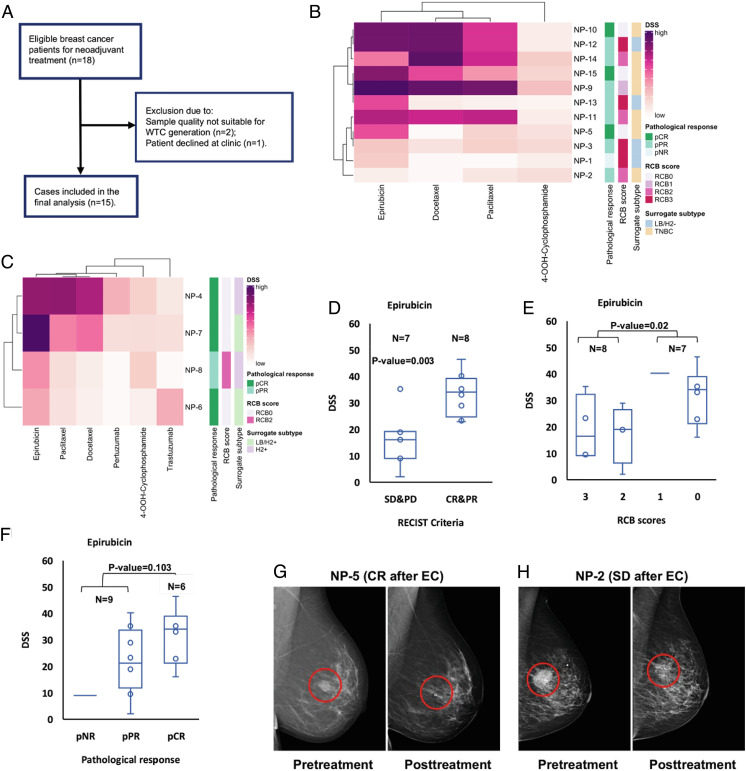
WTC-based drug screening could reflect the clinical outcomes of BC patients. (*A*) The patient inclusion flowchart. (*B*) Heatmap based on the DSSs of established WTCs from the core needle biopsies of LB/H2− and TNBC patients. DSSs were clustered using Ward’s hierarchical clustering algorithm and Spearman’s rank-based correlation coefficient. Cells are color-coded by the DSS value. Rows correspond to individual patients, and columns represent compounds. Samples are annotated with the clinical subtypes of the tumors and their pathological evaluations of neoadjuvant treatment response. (*C*) Heatmap based on the DSSs of established WTCs from the core needle biopsies of LB/H2+ and H2+ patients. DSSs were clustered using Ward’s hierarchical clustering algorithm and Spearman’s rank-based correlation coefficient. Cells are color-coded by the DSS value. Rows correspond to individual patients, and columns represent compounds. Samples are annotated with the clinical subtypes of the tumors and their pathological evaluations of neoadjuvant treatment response. (*D*) Box plot of the epirubicin DSSs for 15 patients in the SD/PD group (N = 7) versus the CR/PR group (N = 8) according to RESIST guideline (RESIST 1.1). Wilcoxon–Mann–Whitney test *P*-value = 0.003. (*E*) Box plot of the epirubicin DSSs for 15 patients in relation to the RCB scores of their residual tumors. Wilcoxon–Mann–Whitney test between RCB scores 0 & 1 (N = 7) versus 2 & 3 (N = 8) groups *P*-value = 0.02. (*F*) Box plot of the epirubicin DSSs for 15 patients in relation to the pathological response evaluations of their residual tumors. Wilcoxon–Mann–Whitney test between pNR & pPR (N = 9) versus pCR (N = 6) groups *P*-value = 0.103. (*G*) Selected mammography images of NP-5 tumor before and after neoadjuvant treatment with the EC regimen. Red circles indicate the tumor area. (*H*) Selected mammography images of NP-2 tumor before and after neoadjuvant treatment with the EC regimen. Red circles indicate the tumor area. EC: epirubicin + cyclophosphamide; CR: complete response; PR: partial response; RCB: residual cancer burden; SD: stable disease; PD: progressive disease.

We next examined the consistency between WTC-based drug profiling results and BC patients’ clinical responses in the neoadjuvant setting. A total number of 18 primary BC patients were recruited by their responsible clinicians. One additional core needle biopsy was taken from individual patients for WTC cultivation and drug testing before initiation of neoadjuvant treatment. Eventually, 15 patients were included in the validation study (sufficient material could not be generated from two patients and one patient declined inclusion) (Fig. 6*A*).

Clinically, epirubicin and cyclophosphamide (EC) were first given to all the patients for three or four cycles, followed by either docetaxel or paclitaxel. The HER2-positive patients also received trastuzumab and pertuzumab together with the taxane treatment. After neoadjuvant treatment, six out of 15 patients achieved pathological complete response (pCR) and residual cancer burden (RCB) score 0; eight patients achieved pathological partial response (pPR) and RCB score ranging from one to three; one patient had no pathological response (pNR) with RCB score 3 (32, 33) (*SI Appendix*, List S1).

According to the WTC-based testing, all the LB/H2- and TNBC patients eventually achieved pCR (three out of 11) showed mid-to-high DSSs for epirubicin, and radiology assessment showed that their tumors had disappeared even before the initiation of taxane treatment (Fig. 6 *B* and *G* and *SI Appendix*, Fig. S8*H*, List S1). A higher proportion of LB/H2+ and H2+ patients ultimately achieved pCR (three out of four), though our data suggested it was likely a combinational effect of all medications since only one patient’s (NP-4) tumor became undetectable by just receiving EC (Fig. 6*C* and *SI Appendix*, Fig. S8*J*). Moreover, the only HER2-positive patient (NP-8) who ended up with pPR also did not show a satisfactory ex vivo response with any of the prescribed regimens (Fig. 6*C*).

Low DSSs of epirubicin was observed among a HER2-negative patients cluster (NP-1 & 2 & 3) who did not achieve pCR at last (Fig. 6 *B* and *H* and *SI Appendix*, Fig. S8*I*), though a few patients with mid-to-high DSSs of epirubicin also only reached pPR eventually. Therefore, we evaluated the possible DSS cutoff of epirubicin according to the Response Evaluation Criteria in Solid Tumors (RECIST) criteria, which is widely used to judge the effectiveness of individual treatment through image-based assessment (34). Tumor sizes before and after EC treatment were measured by a senior breast radiologist, and the clinical responses were defined into complete response (CR) or partial response (PR) as effective, versus stable disease (SD) or progressive disease (PD) as otherwise. The DSS of epirubicin was significantly higher in the response effective patient group with a median of 34.2, versus 16.1 for the ineffective group (Fig. 6*D*). This observation was still consistent when comparing patient groups with RCB scores 0 and 1 versus 2 and 3 (Fig. 6*E*), as well as for patients who finally achieved pCR versus non-pCR (Fig. 6*F*). Noteworthy, the only patient with RCB score 1 had no residual tumor left after pathology analysis, and the treatment eradicated all lymph node metastases except one (*SI Appendix*, List S1). However, no significant difference was found for the DSSs of 4-OOH-cyclophosphamide, suggesting epirubicin was a determinant regimen for patient outcomes in this study (*SI Appendix*, Fig. S8 *A*–*C*). It is worth mentioning that due to the clinical sampling restrictions between treatment switches, the DSSs for regimens other than EC were also calculated based on their testing results from the untreated patient biopsy-derived WTC cells. However, their clinical effectiveness assessments were made on top of the prior EC treatment, which could partly explain the ambiguous findings for the taxane treatment comparisons (*SI Appendix*, Fig. S8 *D*–*G*). Other factors that affected the comparison of docetaxel and paclitaxel were treatment early stops or regimen changes due to severe side effects, as well as tumor HER2 status changes (negative to positive, NP-12) during the treatment period (*SI Appendix*, List S1).

## Discussion

BC studies have generally been carried out with different cell lines grown during 2D culture conditions. Such models are associated with excellent manipulability, reproducibility, and high-throughput screening feasibility. However, they cannot fully represent the complex biology of BC (11). The PDX mouse models, though developed decades ago, are regaining popularity in recent years by their advantages in capturing tumor heterogeneity, representing physiological conditions, and modeling disease development to some extent. However, their poor manipulability and lack of high-throughput screening capability are the major drawbacks that significantly impede their routine clinical utility, not to mention the low tumorigenicity for BC inoculation (14, 35–37). The breakthrough of organoid technology during the last 10 y has equipped us with new tools for studying cancer epithelial cells by closely mirroring the in vivo milieu (38–40). While the tumor organoid models allow efficient ex vivo screening of drug responses for individual patients, limitations such as the risk of selective clonal expansion, time-consuming and costly to culture, unforeseeable growth kinetics, and lack of the TME consideration are also unneglectable (41). The TME harbors various immune and non-immune stromal cell populations, which contribute to tumorigenesis and influence patients’ therapeutic responses (42). For instance, the cancer-associated fibroblasts (CAFs) have been indicated for their roles in the elevation of tumor cell stemness features and acquisition of chemoresistance for BC (43, 44), as well as being the determinants of BC molecular subtypes that could therefore impact the therapeutic effects (45). Also, the infiltration of immune cells within the tumor milieu has been suggested for its prognostic values (46). Moreover, a stromal gene signature was identified for the prediction of BC patient outcome independent of tumor subtype (47).

In this study, we described that patient-derived WTC efficiently represents the parental tumor, and therefore serves as a suitable ex vivo model to facilitate BC precision oncology studies. Compared with the epithelial-only organoid models (15, 48), or coculture of peripheral blood cells with organoids (49), the BC WTCs preserve the primary tumor cells with their endogenous TME components (Fig. 1 *D*–*F* and *SI Appendix*, Fig. S1*B*). By applying transcriptome analysis (Fig. 3*C*), we also presented the interpatient heterogeneity of stromal cell populations, which can affect individual treatment responses. Furthermore, we were able to generate the WTC and score the drug sensitivities for individual patients within 10 d, whereas the cultivation of organoids can take up to a few months, and that is an unrealistic time scenario for the decision of treatment regimens in clinical routine.

Additionally, one of the major challenges for establishing patient-derived ex vivo models is always the availability and accessibility of tumor samples. BC screening results in earlier diagnosis and hence, smaller tumors. Take Sweden as an example, the average tumor size is currently 15 mm in diameter (50), which often makes it difficult to sample research materials without obstructing routine diagnostics. However, by incorporating previously developed superficial scraping techniques (51), we could raise the research sample inclusion rate to above 75% for BC patients enrolled in our research program. It is of great importance for patients whose disease is diagnosed at the early stages or for patients who only achieve pPR after neoadjuvant therapy and need further adjuvant treatment.

Genomic aberrations are the driving force of cancer cell growth. While the NGS techniques have notably advanced our knowledge of cancer genetics, our options for efficient treatments targeting these alterations still lag behind. Not to mention the significant limitations of current strategies by sequencing only a fraction of the coding regions. A recent study reported that only 19% of successfully sequenced tumors were matched with targeted treatments in a cohort of 843 patients with advanced diseases, while the ratio of patients who eventually benefited from this approach was barely 7% (52). Therefore, more levels of cellular information are essential to decipher the cancer malignant mechanisms, as well as to tailor more individualized treatments.

The current systemic therapies for BC include endocrine therapy, chemotherapy, targeted therapy, and a few emerging immunotherapy options. In this study, we described the unique methodology of combining WTC treatment and RNA sequencing for testing individual patient sensitivity toward endocrine therapy, where the tamoxifen active metabolites 4OHT and EDF were studied. We successfully discovered a positive correlation between the expected response to endocrine treatments with tumor *ESR1* transcript abundance (Fig. 4*C*). Interestingly, the patient RNA-13 was identified as an outlier of the group trend though presenting high levels of *ESR1* transcripts, which suggested that the patient could be a nonresponder to tamoxifen treatment. Of note, the tumor of patient RNA-1 had only 20% of ER-positive cells, as well as a low abundance of *ESR1* and *PGR* transcripts, which could explain its poor response to endocrine treatment (*SI Appendix*, List S1). Additionally, although a few EDF-focused clinical trials have been carried out and emerging pieces of evidence have been collected to support its promising antitumor activity, most of them are still ongoing and the molecular basis of EDF compared to 4OHT in vitro is not yet fully understood (25, 53). Contributing to these earlier explorations, our data show that the majority of WTCs exhibited the same responsive trend to 4OHT and EDF treatment. Our finding is in line with recent studies applying EDF as replacement therapy for patients who are deficient in metabolizing tamoxifen citrate due to their variant form of the *CYP2D6* enzyme (25, 54). This is also advocated by our GSEA analysis using the Hallmarks and Canonical Pathways gene-set collections, where both treatments triggered similar molecular pathway events (Fig. 4 *A* and *B* and *SI Appendix*, Fig. S3 *B* and *C* and Tables S3–S6).

We also investigated the responses of WTCs to BC-relevant chemotherapies and targeted therapies through a comprehensive ex vivo drug profiling. With a well-established DSS calculation pipeline and unsupervised clustering analysis, we observed that drugs with a similar mechanism of action were naturally clustered together, as well as distinct responses of individual patients toward individual compounds. We found several interesting associations between the DSS data and biomarker status in our drug-profiled tumors, where expected drug efficacies were indeed achieved significantly better within criteria-eligible patient groups (*SI Appendix*, Figs. S5 *B*–*E* and S6 *A*–*H*). For instance, *BCL2* is an estrogen-regulated gene and is overexpressed in around 85% of ER-positive breast tumors (55). The expression of BCL-2 has been reported to have clinical importance in primary BC where it positively correlates with prognostic factors such as ER/PR expression, HER2 negativity, slow proliferation, and low tumor grade (56, 57). Besides, the beneficial effects of venetoclax were observed among ER-positive primary BC PDX mouse models (58). Therefore, our noteworthy findings on venetoclax are largely in parallel with the previous knowledge (Fig. 5*D* and *SI Appendix*, Figs. S5 *B* and *C*, S6*H*). Additionally, the use of venetoclax as a drug for ER-positive HER2-negative locally advanced or metastatic BC patients, together with endocrine therapies, is currently being studied clinically with initial supportive findings (59). Moreover, we have also successfully utilized WTC-based drug testing assays earlier to study the tumor-suppressive effects of anticancer compound RITA (Reactivation of p53 and Induction of Tumor cell Apoptosis), aminoflavone (AF), and oncrasin-1 (ONC-1), in combination with poly(ADP-ribose) polymerase (PARP) inhibitors or 4-hydroxy-tamoxifen (60, 61).

Finally, we evaluated the WTC model and its utility for decision support through a neoadjuvant validation study. We found that our ex vivo profiling data were in line with the patient eventual clinical responses to a large extent, particularly for epirubicin and anti-HER2 dual inhibitors. We were able to identify epirubicin as the decisive regimen for patient outcomes in this study and provided distinct DSS reference ranges. Collectively, these findings highlighted the potential of WTCs in facilitating BC patients’ treatment decisions, patient assignment to appropriate clinical trials, and the validation of potential biomarkers during drug development.

The WTC model has its limitations as well. Long-term culture and passage can be difficult, especially for the maintenance of various stromal cell populations. Besides, the lack of complete tumoral vasculature and physical barriers make it difficult to test the response of some therapies, such as angiogenesis-targeted and immunomodulatory drugs, as well as hard to elucidate the difference in drug delivery. Despite these current weaknesses, our results suggest the strong clinical association and predictive value of WTCs for endocrine therapy, chemotherapy, and targeted therapy regimens for BC patients. Additionally, the WTC model showed the capacity to perform medium- to high-throughput drug screenings efficiently. Therefore, we would propose the BC WTC as an advantageous preclinical model for academic research and pharmaceutical drug development, also with good potential to assist future patient treatment design after further clinical validation studies.

## Materials and Methods

### Approval and Collection of Clinical Tumor Material.

Fresh samples from surgically resected breast tumor specimens were obtained from the Department of Clinical Pathology and Cancer Diagnostics at Karolinska University Hospital, Stockholm, Sweden. The core needle biopsies in the validation study were collected at the breast center of Stockholm South General Hospital (Södersjukhuset). Experimental procedures and protocols were approved by the regional ethics review board (Etikprövningsnämnden) in Stockholm. Detailed information can be found in the *SI Appendix*, *Materials and Methods*.

### BC WTC.

Original BC TSCs were collected by superficial scraping as previously reported (51). Upon arrival at the lab, the TSCs were washed, digested with Dispase, and filtered before culturing. For the needle biopsies, the tissues were first homogenized for cell release, washed, and filtered before culture. The detailed sample processing protocol and WTC culture conditions are described in the *SI Appendix*, *Materials and Methods*.

### WGS.

A normal skin biopsy for germline control, TSCs collected at the time of surgical resection and derived WTC cultures were collected from each patient's specimen. Genomic DNA samples were isolated using the QIAamp DNA mini kit (QIAGEN). The library was prepared using Illumina TruSeq PCR-free (350 bp) according to the manufacturer’s protocol. The bulk DNA samples were then sequenced by Illumina Hiseq X and processed via the Science for Life Laboratory CAW workflow version 1.2.3 (62) (Stockholm, Sweden; https://github.com/SciLifeLab/Sarek). Briefly, following the GATK best practices preprocessing steps, each sample was aligned using Burrows–Wheeler Aligner (BWA) MEM algorithm version 0.7.16a (63) to the human genome assembly (build GRCh38), followed by duplicate marking and base recalibration (64). The methods used for somatic SNV and indel calling, copy number analysis, gene annotation, and figure plotting are described in the *SI Appendix*, *Materials and Methods*.

### RNA Sequencing.

The TSC pellets from the time of surgical resection, and the pellets of derived WTC cultures with or without tamoxifen metabolites treatment (1 nM 4OHT and 25 nM Z-EDF) (65) were collected. Around 2,000 ng RNA was extracted using the RNeasy mini kit (QIAGEN) from each sample, and 1 μg total RNA was used for rRNA depletion using RiboZero (Illumina). Stranded RNAseq libraries were constructed using TruSeq Stranded Total RNA Library Prep Kit (Illumina), and paired-end sequencing was performed on HiSeq 2500 with a 2 × 126 setup using the Science for Life Laboratory platform (Stockholm, Sweden). The detailed analysis methodology of the RNA sequencing data can be found in the *SI Appendix*, *Materials and Methods*.

### Cell Viability Assay.

All the compounds were stored and prepared according to the manufacturer's recommendation. They were dispensed with liquid handling systems to make spotted 384-well drug plates covering concentrations between 10 μM to 1 nM for each drug (2 μM to 0.2 nM for trastuzumab and pertuzumab). Detailed purchase information for all the compounds can be found in the *SI Appendix*, List S3.

The patient-derived WTC spheroids were dissociated, filtered, and resuspended as single cells for the assay. Afterward, 2 × 10^3^ cells/well were transferred into the drug-spotted plate using a digital dispenser and moved to a cell culture incubator. The viability was assessed after 96 h, and the DSSs were calculated. A comprehensive description of the drug profiling assay and DSS analysis method can be found in the *SI Appendix*, *Materials and Methods*.

### NanoString nCounter® BC 360 Panel.

HE slides corresponding to the FFPE tumor tissue samples of WTC drug-profiled patients were reviewed by a breast pathologist. Areas containing viable invasive tumor cells were marked, and 10-μm sections were macrodissected from the FFPE tissue samples accordingly. RNA was extracted from the macrodissected sections using the High Pure FFPET RNA Isolation Kit (Roche) following the manufacturer’s protocols. Then, 200 ng RNA per sample was loaded and further analyzed according to the manufacturer’s recommendation on a NanoString nCounter**®** system using the BC 360 code set, which is comprised of 18 housekeeping genes and 752 target genes covering key pathways in tumor biology, microenvironment, and immune response. The detailed analysis method can be found in the *SI Appendix*, *Materials and Methods*.

The detailed methods of IHC, immunofluorescence staining, flow cytometry, and real-time Imaging are described in the *SI Appendix*, *Materials and Methods*.

## Supplementary Material

Appendix 01 (PDF)

## Data Availability

The study's original data (WGS, RNA-seq, BC360 panel, and cell viability assay) are deposited on a secure Swedish server and has been assigned a DOI (https://doi.org/10.17044/scilifelab.21516993). Data access requests may be submitted to the Science for Life Laboratory Data Centre through the DOI link. All other data are included in the article and/or *SI Appendix*. Scripts to reproduce the WGS and RNA-seq analysis as part of the study are available at: https://doi.org/10.17044/scilifelab.21647783 (66, 67).
