# Supplementary material for: Breast cancer patient-derived whole-tumor cell culture model for efficient drug profiling and treatment response prediction
Source: Proc Natl Acad Sci U S A. 2022 Dec 27;120(1):e2209856120. doi: 10.1073/pnas.2209856120 (PMC9910599; doi:10.1073/pnas.2209856120)
Supplement: Supplementary file 1 — Appendix 01 (PDF) [file pnas.2209856120.sapp.pdf]

PNAS

Supporting Information for
Breast cancer patient-derived whole-tumor cell culture model for efficient drug profiling and treatment response prediction

Xinsong Chen, Emmanouil G. Sifakis, Stephanie Robertson, Shi Yong Neo, Seong-Hwan Jun, Le Tong, Apple Hui Min Tay, John Lövrot, Roxanna Hellgren, Sara Margolin, Jonas Bergh, Theodoros Foukakis, Jens Lagergren, Andreas Lundqvist, Ran Ma, and Johan Hartman

Xinsong Chen
Email: xinsong.chen@ki.se
Johan Hartman
Email: johan.hartman@ki.se

This PDF file includes:

Supporting text
Figures S1 to S8
Tables S1 to S6
Lists S1 to S3
SI References

Supporting Information Text

Supplementary Materials and Methods

Approval and collection of clinical tumor material

Fresh samples from surgically resected breast tumor specimens were obtained by the superficial scraping method at the Department of Clinical Pathology and Cancer Diagnostics at Karolinska University Hospital, Stockholm, Sweden between 2016 and 2021. The collected BC TSCs with their later derived WTCs were assigned to different experimental arms randomly, without knowing their clinical characteristics at the time of sampling and experiments. The pathology parameters and other clinical information of all the studied tumors were retrieved after the completion of the experiments. For the validation study cohort, all patients were recruited during 2020 and 2021 with breast tumors naïve to treatment. For this purpose, eligible patients were identified at the tumor board by responsible clinicians before patients were asked for informed consent. From included patients in the validation study, one extra study core needle biopsy was collected by radiologists at the breast center of Stockholm South General Hospital (Södersjukhuset). Experimental procedures and protocols were approved by the regional ethics review board (Etikprövningsnämnden) in Stockholm, with reference numbers 2016/957-31, 2017/742-32, 2020-00323, and 2021-00795. Biobank approval was obtained from the Stockholm medical biobank. Before surgery or neoadjuvant treatment, informed consent in accordance with the Declaration of Helsinki was given to patients for signature. Clinical information for each patient specimen involved in this study, as well as the individual treatment details for the validation cohort patients, can be found in List S1.

Breast cancer whole-tumor cell culture

The TSCs were collected by pathologists and placed in cold DMEM-F12 GlutaMAX supplement medium (31331-028, ThermoFisher Scientific) tubes. Upon arrival at the lab, the tube was centrifuged (300 x g, 4 °C) to remove the medium, followed by two repeats of DPBS (14190144, ThermoFisher Scientific) washing steps. The final pellet was gently disturbed by Dispase (07923, Stemcell Technologies) for 5-10 minutes at 37 °C, followed by passing through a 100 mm nylon strainer (Miltenyi Biotec). After rinsing off Dispase by washing another two times with DPBS, all the

cells were placed in the Millicell® cell culture inserts (PICM03050 or PICM01250, 0.4-μm pores Teflon membrane, Millipore) and maintained in 6 or 24-well plates (CLS3516 or CLS3526, Corning), supplied with DMEM-F12 GlutaMAX supplement medium including 10 mM HEPES buffer solution (15630-056, Gibco), 10% inactivated FBS (10500-064, Gibco), 1× non-essential amino acids (11140-035, Gibco), 40 ng/ml human recombinant EGF (78136, Stemcell Technologies), 20 ng/ml human recombinant FGF (100-AF-18B, PeproTech), and 1x Primocin (ant-pm-1, InvivoGen). From the needle biopsies, the tissues were homogenized and the cells were released by using the gentleMACS™ Octo Dissociator with heaters and the human tumor dissociation kit (both from Miltenyi Biotec), according to the manufacturer´s protocols. Afterward, the cells were washed two times with DMEM-F12 GlutaMAX supplement medium and collected by centrifugation at 300 x g for 7 minutes. The WTCs were further generated by passing the re-suspended cells through the 100 mm cell strainers, followed by the same culture conditions described above.

Immunohistochemistry

The TSCs and WTCs were fixed in 4% formaldehyde (F8775, SigmaAldrich), paraffin-embedded, sectioned, and subjected to immunohistochemical and hematoxylin-eosin (HE) staining. All stainings were performed at the clinically accredited, routine-pathology laboratory at the Department of Clinical Pathology and Cancer Diagnostics, Karolinska University Hospital. Sections were conditioned in CC1 solution and incubated with monoclonal antibodies for ER (clone SP1, rabbit), PR (clone 1E2, rabbit), HER2 (clone 4B5, rabbit), and Ki67 (clone 30-9, mouse) for staining with the Ventana BenchMark ULTRA IHC/ISH System according to the manufacturer's instructions (all from Roche/Ventana Medical Systems, Tucson, AZ, USA). Finally, the stained sections were counterstained with hematoxylin and mounted. Board-certified pathologists at Karolinska University Hospital assessed each slide and scored ER, PR, HER2, and Ki67 on whole section slides.

Immunofluorescence Staining

The WTCs were fixed with 4% paraformaldehyde (P6148, SigmaAldrich) for 1 hour at 4°C, followed by blocking overnight in DPBS with 0.2% Triton X-100 (T8787, SigmaAldrich), 10% DMSO (D2650-100ML, SigmaAldrich), and 6% Bovine Serum Albumin (BSA, A9647, SigmaAldrich) on a shaker

at 37°C. Samples were then further incubated with primary antibodies diluted in DPBS supplemented with 5% DMSO and 3% BSA (1:200 Mouse Anti-CD45 Antibody, ab8216, Abcam; 1:200 Rabbit Anti-E-cadherin Antibody, sc-7870, Santa Cruz) at 37°C for 24 hrs. After washing with DPBS 3 times, samples were incubated with secondary antibodies (1:200 Alexa 647 donkey anti-mouse IgG H+L antibody, A31571, ThermoFisher Scientific; 1:200 Alexa 555 donkey anti-rabbit IgG H+L antibody, A31572, ThermoFisher Scientific) at 37°C for 24 hrs. After washing with DPBS another 3 times, the samples were again incubated with Human Fibroblast Activation Protein alpha/FAP Alexa Fluor® 488-conjugated Antibody (1:50, FAB3715G, R&D Systems) at 37°C for 24 hrs. Afterward, WTCs were washed in DPBS for 3 times more and incubated with NucBlue™ Live ReadyProbes™ Reagent (R37605, ThermoFisher Scientific) at room temperature for 20 mins and washed again with DPBS. The images were obtained by using a ZEISS LSM 880 microscope.

Flow cytometry

The TSCs and WTCs samples were first washed in PBS, followed by gentle digestion with Dispase at 37°C for 5-10 mins, and passed through a 70 mm nylon strainer (Miltenyi Biotec) for the generation of single-cell suspension. Cells were then re-suspended in PBS and incubated for 30 mins on ice with Human Fc Receptor Binding Inhibitor (14-9161-73, ThermoFisher Scientific). Cell viability is accessed by LIVE/DEAD Fixable Dead cell stain kit and staining is performed with the antibodies listed in the supplementary file List S2. After staining for 30 mins and subsequent washing steps, samples were analyzed on the LSR II BD flow cytometer (BD Biosciences). Data was then analyzed by FlowJo software (BD Biosciences).

Real-time Imaging

Brightfield and phase-contrast images under the 4X objective were acquired every six hours on the IncuCyte S3 system (Essen BioScience). $1x10^4$ cells were seeded and cultured in an ultra-low attachment 96-well plate (174925, Thermo Fisher Scientific) with the culture medium described above. CellEvent™ Caspase-3/7 Green (C10423, Thermo Fisher Scientific) at 1 μM was also added to observe apoptosis. After four days, chemotherapeutics at 100 nM final concentration

were added along with media top-up. Spheroid invasion analysis was performed using top hat segmentation with IncuCyte analysis software according to the manufacturer´s protocols.

Whole-genome sequencing analysis

The somatic SNVs and Indels were called using MuTect2 version 3.8.0(1) and Strelka2 version 2.8.2(2). The matched normal samples were used to distinguish germline variants from somatic ones. Only the somatic SNVs and indels detected by both MuTect2 and Strelka were included in the analysis as a measure against spurious variants. The variant effect prediction was performed using SnpEff version 4.3(3), and the most deleterious variants were identified and assigned on a per gene basis(4).

The copy number analysis was performed using Control-FREEC version 11.5(5). In detecting the (somatic) copy number alterations (CNAs), the window size was set to 1 Kb, and the matched normal samples were provided as input to Control-FREEC along with the TSCs or WTCs. For each gene, the output by Control-FREEC log2 ratios (between the tumor and matched normal samples) at those bins that overlap with the gene body by at least 1 bp were identified and averaged.

Gene annotations were retrieved from Ensembl (GRCh38.p13) through the R/Bioconductor package biomaRt version 2.34.2(6).

The t-SNE plot was generated using the tsne R package version 0.1.3 and was based on the somatic SNVs and CNAs of the BC-specific driver genes and actionable genes(7, 8).

RNA sequencing analysis

Alignment to the human genome assembly (build GRCh37) was carried out using Tophat aligner version 2.0.4. Merged bam files were sorted, and PCR duplicates were marked using Picard MarkDuplicates version 1.29 (http://broadinstitute.github.io/picard). Gene counts were calculated with HTSeq count version 0.6.1(9) with duplicates included. The RNAseq count data were pre-filtered by only keeping in the analysis of those genes that had > 10 reads per million mapped reads in at least two libraries. Data were then normalized using the TMM method(10) available in the edgeR package version 3.24.3(11).

Differential expression between two conditions was analyzed with the gene-wise negative binomial generalized linear model using the R/Bioconductor edgeR package version 3.24.3(11, 12). Adjustment for differences between patients was made by an additive linear model with patients as the blocking factor. Empirical Bayes methods were used to moderate the degree of dispersions across genes. The tests can be viewed as analogous to (moderated) paired t-tests. Enrichment analysis of the Hallmarks and Canonical pathways gene-set collections in the Molecular Signatures Database (MSigDB; Broad Institute, version 7.1) was performed using the GSEA software version 4.0.3 (Broad Institute) with genes pre-ranked according to scores from the differential expression analysis(13). Multiple testing was controlled by calculating the expected false discovery rate (FDR), according to Benjamini & Hochberg(14). The absolute abundance of eight immune and two stromal cell populations was estimated with Microenvironment Cell Populations-counter (MCP-counter) method(15) using the R package MCPcounter version 1.1.0.

All gene expression analysis was done in R/Bioconductor version 3.5.3 unless otherwise specified.

Cell viability assay analysis

Trastuzumab and pertuzumab were freshly prepared in water according to the manufacturer's instructions. All the other compounds were stored as 10 mM aliquots in DMSO according to the manufacturer's recommendation. For the drug profiling assay, each compound covered five concentrations ranging from 10 μM to 1 nM (2 μM to 0.2 nM for trastuzumab and pertuzumab) in 10-fold dilutions and was dispensed using the acoustic liquid handling system Echo 550 (Labcyte Inc) to make spotted 384-well plates. For the neoadjuvant setting validation assay, we updated the cyclophosphamide into its active metabolite form 4-hydroperoxy cyclophosphamide (4-OOH-cyclophosphamide). Each relevant compound covered eight concentrations ranging from 10 μM to 1 nM (2 μM to 0.2 nM for trastuzumab and pertuzumab) and was dispensed using the Tecan D300e Digital Dispenser (Tecan) to make spotted 384-well plates. In both experiment settings, a total volume of 40 nl of each compound condition was dispensed into each well, for limiting the final DMSO concentration to 0.1% during the treatment period.

After 4-5 days of cultivation, WTC spheroids were dissociated into single cells by Dispase digestion at 37°C and filtration with a 70 µm nylon cell strainer, before being suspended in the freshly prepared culture medium again. Afterward, $2x10^3$ cells in 40ul/well of medium were transferred into the drug-spotted 384-well OptiPlate (6007290, PerkinElmer) using the MultiDrop Combi dispenser (ThermoFisher Scientific). The plates were cultured in an incubator with an extra humidity supply for 96 hours. The cell viability was assessed using CellTiter-Glo® 3D cell viability assay (G9683, Promega) according to the manufacturer's instruction and reading luminescence by a Tecan spark 10M microplate reader (Tecan). Positive controls (benzethonium chloride 500 µM, 53751-50G, SigmaAldrich) and negative controls (DMSO, D2650-100ML, SigmaAldrich) from the plate were used to normalize the data.

Quantitatively scoring of drug sensitivity was estimated using the drug sensitivity score (DSS), which captures and integrates the multiparametric dose-response relationships into a single metric(16). Specifically, the raw dose-response readout for each of the tested drugs was first subjected to quality assessment by two independent observers, and response values with outlying behavior were removed. Outliers were defined as response values with an irregular response pattern, or response values with a significantly higher cell count than their corresponding negative controls (maximum one point per drug-response readout was removed due to the limited number of concentrations; in the rare cases of two or more outlying points, the specific dose-response readout was excluded from downstream analysis). The raw dose-response readout was then normalized in relation to the negative and positive controls (median values used), and the relative inhibition (%) was calculated. The normalized dose-response data were fitted using a non-weighted four-parameter logistic regression function, in a similar way as in(16, 17), and the bottom asymptote of the curve was fixed to zero inhibition for each drug(18). The R function “get.curve.data()” in script “calculation_of_css.Rmd” was used for curve fitting, available from GitHub at(17). The DSS (type 3), which varies between 0 (insensitive) and 100 (highly sensitive), was calculated using the R package DSS version 1.2, as previously described(16). The value of 10% for the drug response's minimum activity level was used.

Statistical analyses to assess the differences in the DSS across patients over known biomarkers were performed using either a Kruskal-Wallis test or a Wilcoxon-Mann-Whitney test. P-values were adjusted using the Benjamini & Hochberg procedure(14) (function "p.adjust" in R package stats version 3.4.0) to account for multiple comparisons. Differences were considered significant if p-value < 0.05 unless otherwise stated. All analyses were performed in R version 3.4.0 (http://www.r-project.org/) unless otherwise specified.

The cell viability assay and DSS calculation for the validation cohort patient samples were performed by researchers, and the clinical efficacy was independently evaluated by clinicians. The researchers performing the drug profiling were blinded to the interventions and clinical outcomes of the patients during the treatment period.

NanoString nCounter® Breast Cancer 360 Panel analysis

The raw data of the assay was assessed using several quality assurance (QA) metrics to measure imaging quality, oversaturation, and overall signal-to-noise ratio. All samples satisfying QA metric checks were background corrected (background thresholding) using the negative probes and normalized with their mean minus two standard deviations. The background-corrected data were then normalized by calculating the geometric mean of five housekeeper genes, namely ACTB, MRPL19, PSMC4, RPLP0, and SF3A1(19). Pathway scoring was then performed on the pre-normalized data, where pathway-level information from a group of genes was extracted using the first principal component of their expression data(20).

Both the QA and data pre-processing were done in the nSolver Analysis Software version 4.0 (NanoString Technologies). Pathway scoring was done using nSolver's Advanced Analysis Module version 2.0.134. The normalized nCounter data and the pathway scores were imported into the R computing environment version 3.6.2 for further processing and visualization.

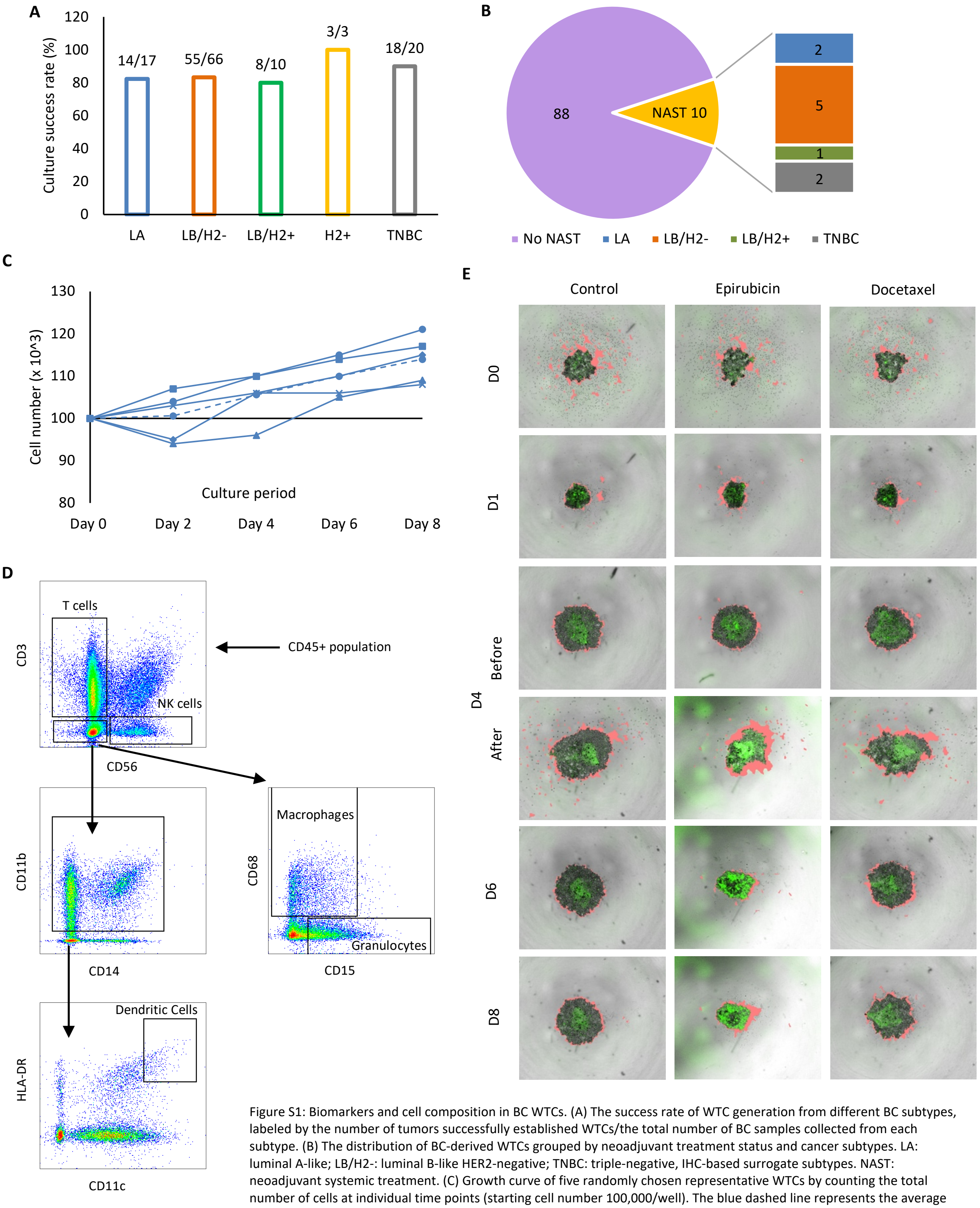

Figure S1: Biomarkers and cell composition in BC WTCs. (A) The success rate of WTC generation from different BC subtypes, labeled by the number of tumors successfully established WTCs/the total number of BC samples collected from each subtype. (B) The distribution of BC-derived WTCs grouped by neoadjuvant treatment status and cancer subtypes. LA: luminal A-like; LB/H2-: luminal B-like HER2-negative; TNBC: triple-negative, IHC-based surrogate subtypes. NAST: neoadjuvant systemic treatment. (C) Growth curve of five randomly chosen representative WTCs by counting the total number of cells at individual time points (starting cell number 100,000/well). The blue dashed line represents the average curve of the five WTCs (2 TNBC, 2 LB/H2-, 1 LB/H2+, all without NAST). (D) Representative flow cytometry analysis chart of various immune cell (CD45+) proportions within the WTC cultures. (E) Representative real-time imaging of WTC cultures over 8 days under 4X objective, with untreated control (first column) or added chemotherapeutics indicated at day 4. The red mask demarcates the area with cells surrounding the WTC spheroid, while the light green area indicates Caspase-3/7 levels (Before: prior to the addition of epirubicin and docetaxel; After: subsequent to the addition of epirubicin and docetaxel).

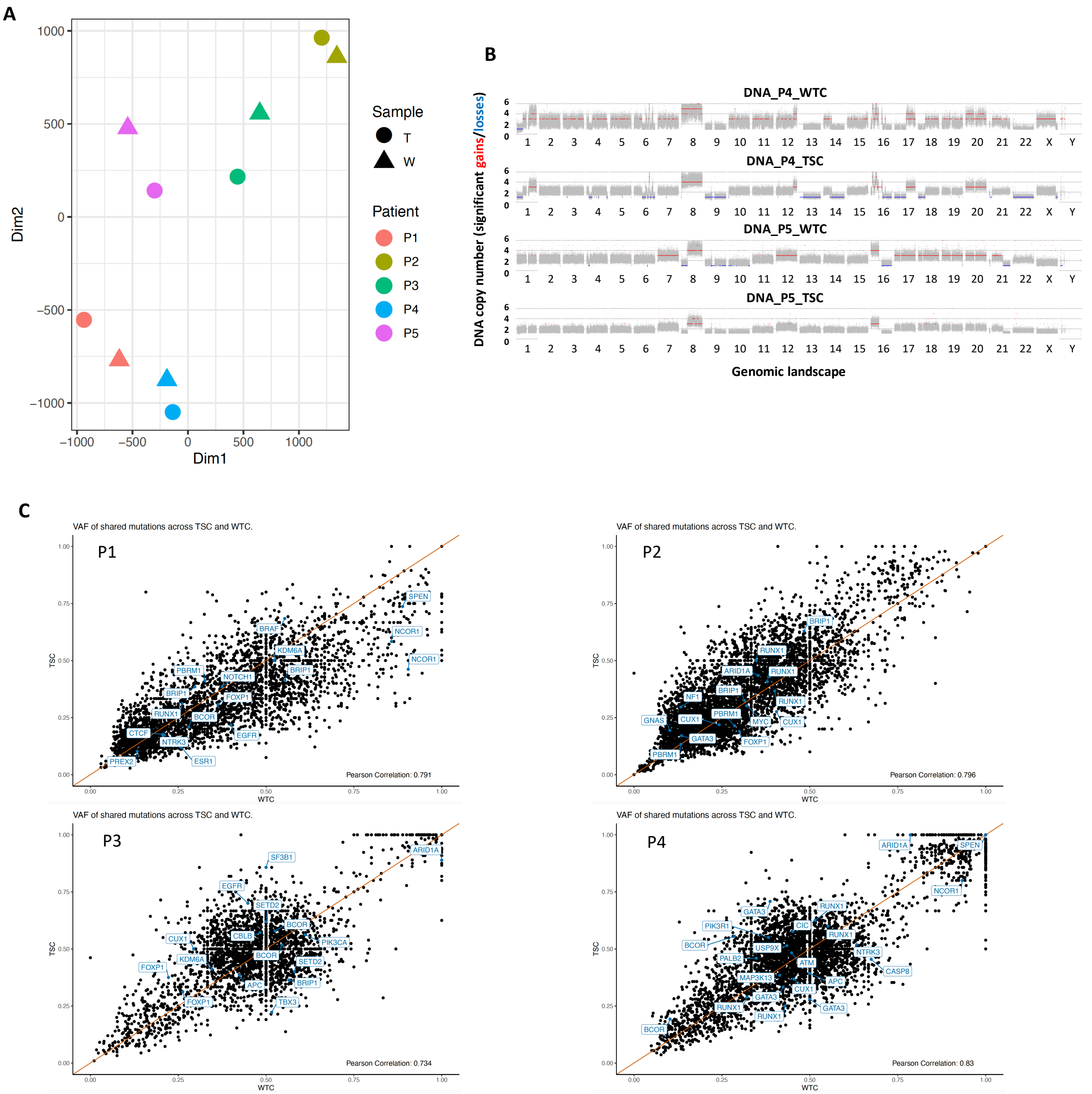

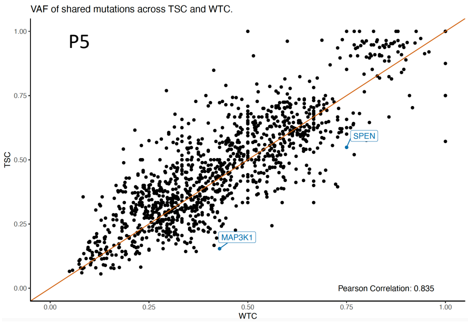

Figure S2: Patient-sample projection in a two-dimensional map illustrating the similarity of the TSC-WTC pairs and CNA profile analysis of BC WTCs. (A) t-distributed stochastic neighbor embedding (t-SNE) plot of the five TSC (T) and WTC (W) pairs. Each point, either in a triangle (T) or square (W), represents a sample genomic profile based on the somatic SNVs and CNAs of the BC-specific driver genes and actionable genes. Patients are labeled by color. (B) CNAs patterns between BC TSCs and established WTCs from additional patients. (C) Scatterplot for comparing VAF values of all the shared mutations between TSC and WTC pairs of individual patients. The mutations found in BC-specific driver genes and actionable genes are highlighted in blue color and labeled with names. The Pearson correlation method is used for measuring the linear relationship (concordance).

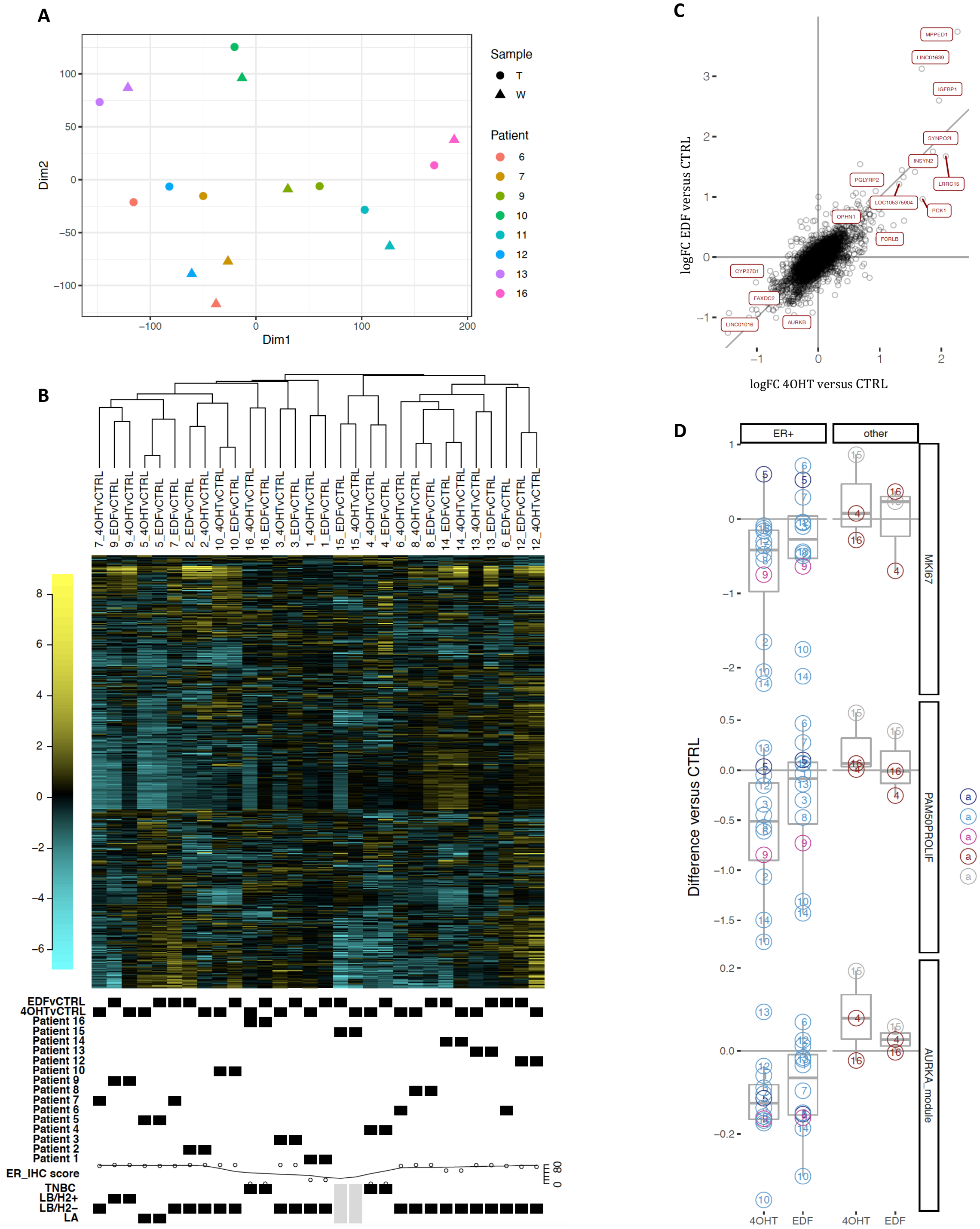

Figure S3: BC WTCs allow *ex vivo* evaluation of patient-specific sensitivity to endocrine therapy by RNA sequencing. (A) t-distributed stochastic neighbor embedding (t-SNE) plot of the TSC (T) and WTC (W) sample pairs. Each point, either in a triangle (T) or square (W), represents a sample transcriptomic profile based on the global RNA expression patterns. Patients are labeled by color. (B) Unsupervised clustering of the pair-wise differences based on the top 500 most varying transcripts. Specifically, average-linkage hierarchical clustering was performed with the one minus correlation metric. The heatmap cells are color-coded in comparison to the gene expression in both 4OHT and EDF treatments versus the untreated condition (CTRL) (yellow: higher than overall gene expression in untreated condition; black: same as in untreated condition; blue: lower than in untreated condition). Annotation rows show patient ID, conditions compared (treatment with 4OHT/EDF vs. untreated), ER value, and clinical IHC-based surrogate subtype assignment on surgical specimen. (C) Scatterplot of the logFCs for the two treatment comparisons. The top 10 differentially abundant transcripts in any of the comparisons are marked with their gene name. logFC: log2-fold change. (D) Changes in *MKI67* transcript abundances, PAM50 proliferation index, and AURKA module score as the surrogate markers of treatment response. Numbers in circles correspond to patient IDs, color-coded based on the clinical surrogate subtype assignment on surgical specimen. Overlaid box plots indicate the median and interquartile range. LA: luminal A-like; LB/H2-: luminal B-like HER2-negative; LB/H2+: luminal B-like HER2-positive; TNBC: triple-negative; NA: not applicable.

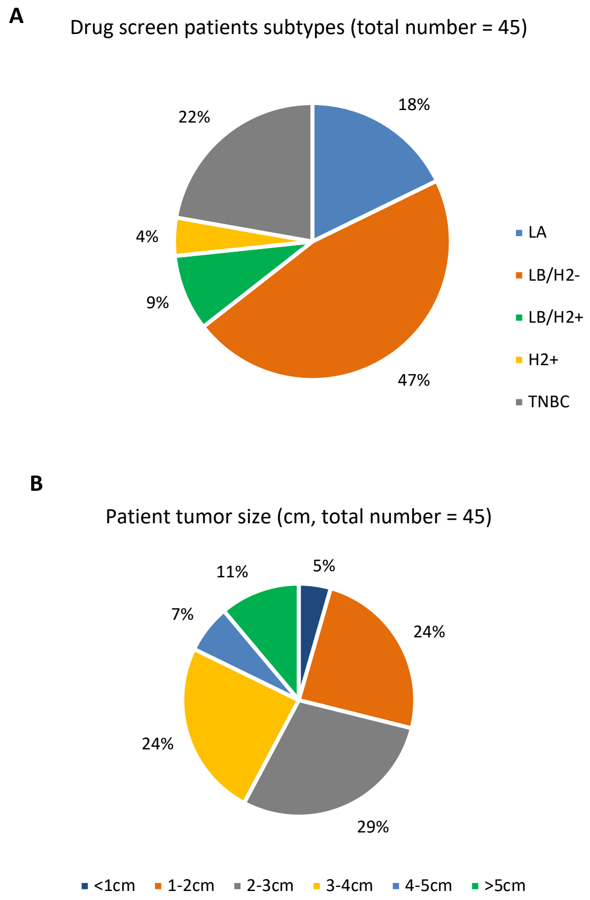

Figure S4: Clinical characteristics of drug profiled BC WTCs. (A) Clinical IHC-based surrogate subtypes and (B) size distributions of the original BC patient tumors from which we have established WTCs for drug profiling. LA: luminal A-like; LB/H2-: luminal B-like HER2-negative; LB/H2+: luminal B-like HER2-positive; H2+: HER2-positive non-luminal; TNBC: triple-negative.

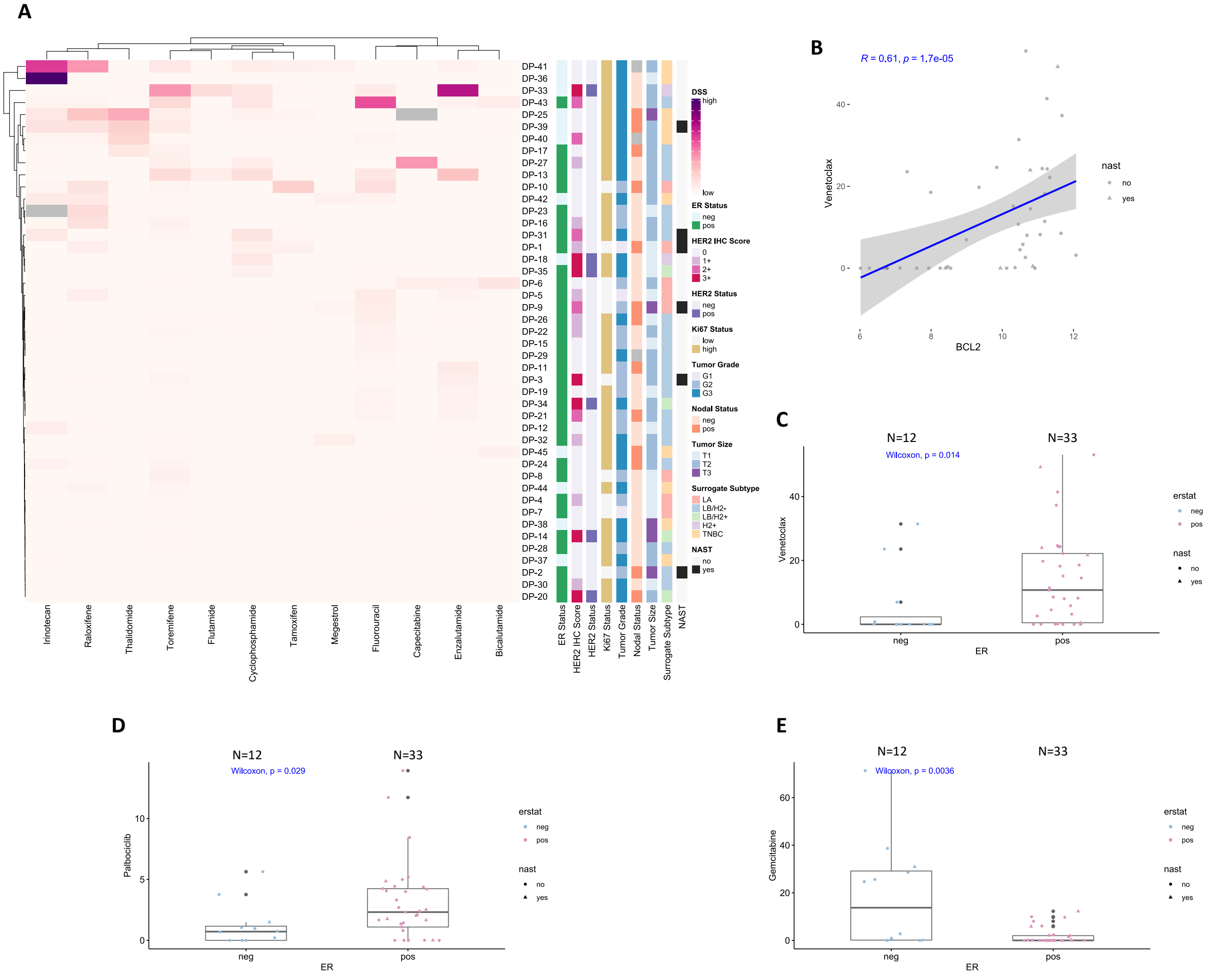

Figure S5: BC WTCs allow *ex vivo* drug screening. (A) Heatmap based on the DSSs from established WTCs only for the liver metabolism-dependent compounds. DSSs were clustered using Ward's hierarchical clustering algorithm and Spearman's rank-based correlation coefficient. Cells are color-coded by the DSS value (light gray color indicates not available data). Rows correspond to individual patients, and columns represent compounds. Samples are annotated with the clinical characteristics of the original breast tumors. LA: luminal A-like; LB/H2-: luminal B-like HER2-negative; LB/H2+: luminal B-like HER2-positive; H2+: HER2-positive non-luminal; TNBC: triple-negative, IHC-based surrogate subtypes. NAST: neoadjuvant systemic treatment. (B) Correlation analysis between *BCL2* mRNA expression levels (log2-transformed and normalized counts) and venetoclax DSS across individual patients (Spearman's rank correlation coefficient =0.61, p-value=1.7e-05). The straight blue line represents the best fit (linear regression) to the data, and the gray band around the line indicates a 95% confidence level. Associations of ER status and distribution of DSS for (C) venetoclax (neg: N=12, pos: N=33, Wilcoxon-Mann-Whitney test p-value=0.014), (D) palbociclib (neg: N=12, pos: N=33, Wilcoxon-Mann-Whitney test p-value=0.029) and (E) gemcitabine (neg: N=12, pos: N=33, Wilcoxon-Mann-Whitney test p-value=0.0036). Box-and-whisker plots indicating the median and interquartile range are overlaid on the strip charts. pos: ER staining positivity ≥ 1%; neg: ER staining positivity < 1%.

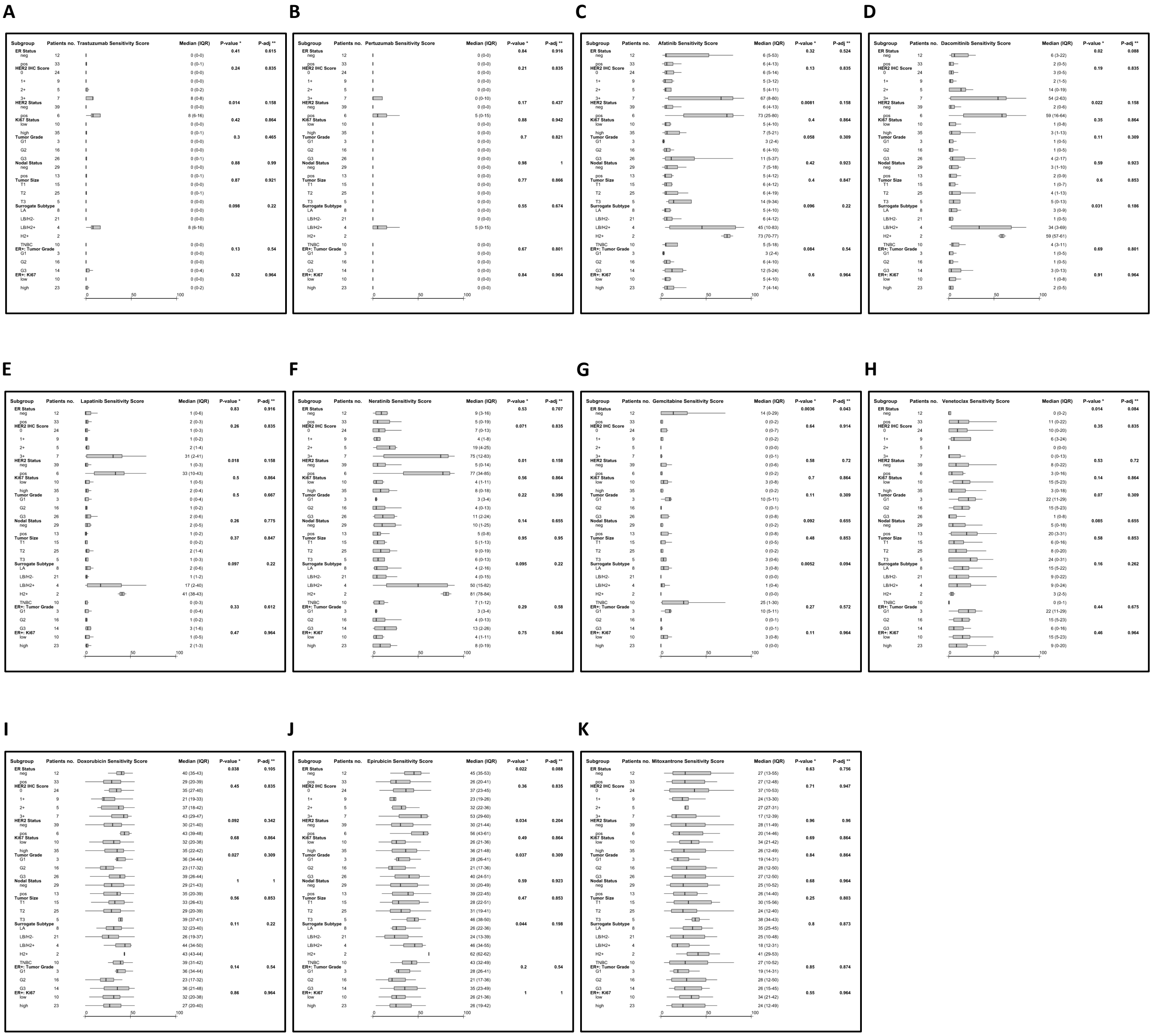

Figure S6: Associations of various patients' clinicopathological characteristics and distribution of DSS. (A) trastuzumab, (B) pertuzumab, (C) afatinib, (D) dacomitinib, (E) lapatinib, (F) neratinib, (G) gemcitabine, (H) venetoclax, (I) doxorubicin, (J) epirubicin and (K) mitoxantrone. Box-and-whisker plots indicate the median and interquartile range (IQR). LA: luminal A-like; LB/H2-: luminal B-like HER2-negative; LB/H2+: luminal B-like HER2-positive; H2+: HER2-positive non-luminal; TNBC: triple-negative, IHC-based surrogate subtypes. * Wilcoxon-Mann-Whitney or Kruskal-Wallis test. ** Benjamini & Hochberg adjusted p-value.

A

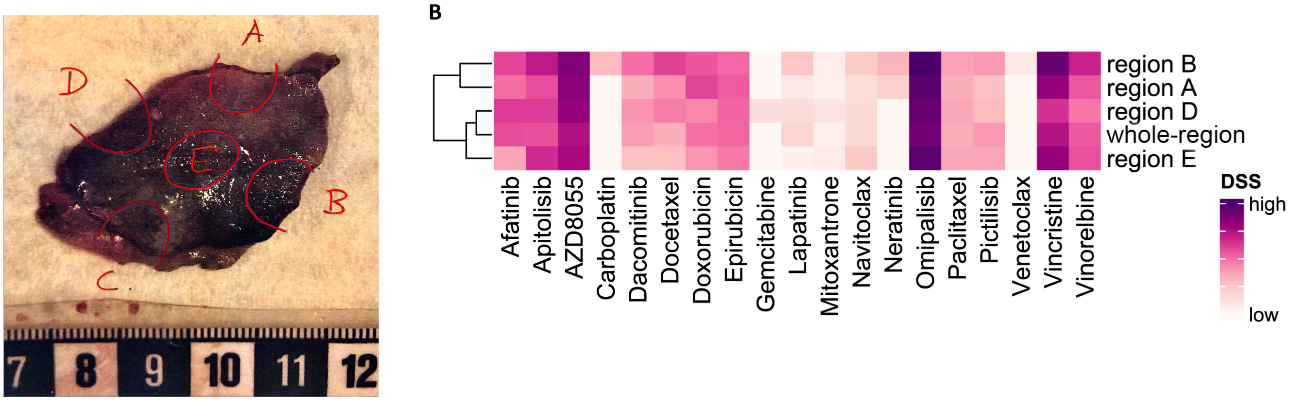

Figure S7: Drug profiling for studying intra tumoral heterogeneity. (A) Multiple regions of a TNBC tumor DP-45 were sampled to establish WTCs and perform drug profiling. Region C was omitted due to an insufficient number of cells. (B) Heatmap based on the DSSs from established WTCs only for compounds that have shown responsiveness in at least one of the regions of tumor DP-45. DSSs were clustered using Ward's hierarchical clustering algorithm and Spearman's rank-based correlation coefficient. Cells are color-coded by the DSS value. Rows correspond either to individual tumor regions or the whole tumor region, and columns represent compounds.

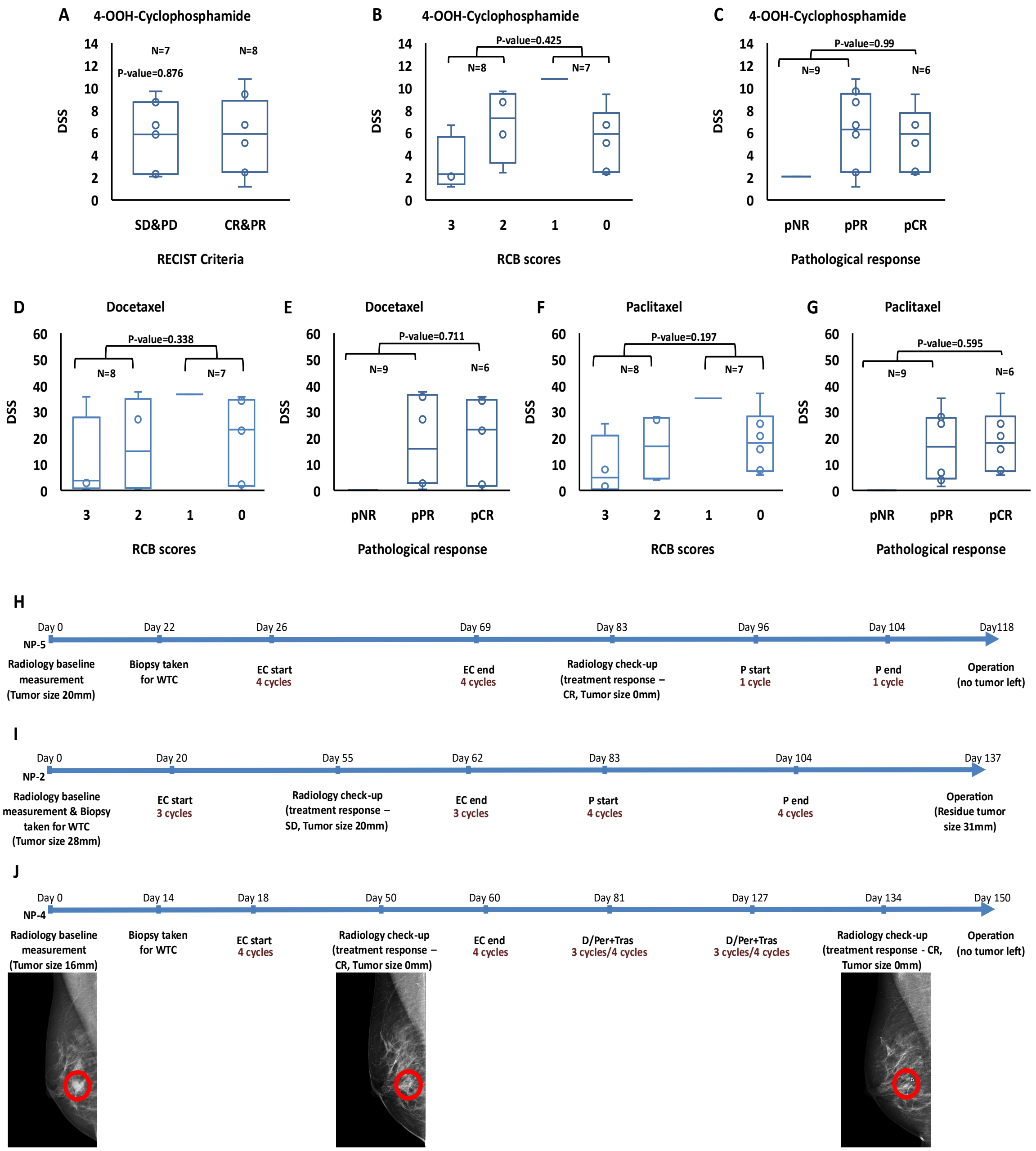

Figure S8: WTCs based drug screening could reflect the clinical outcomes of breast cancer patients. (A) Box plot of the 4-OOH-cyclophosphamide DSSs for 15 patients in the SD/PD group (N=7) versus the CR/PR group (N=8) according to RESIST guideline (RESIST 1.1). Wilcoxon-Mann-Whitney test p-value=0.876. (B) Box plot of the 4-OOH-cyclophosphamide DSSs for 15 patients in relation to the RCB scores of their residual tumors. Wilcoxon-Mann-Whitney test between RCB scores 0&1 (N=7) versus 2&3 (N=8) groups p-value=0.425. (C) Box plot of the 4-OOH-cyclophosphamide DSSs for 15 patients in relation to the pathological response evaluations of their residual tumors. Wilcoxon-Mann-Whitney test between pNR&pPR(N=9) versus pCR (N=6) groups p-value=0.99. (D) Box plot of the docetaxel DSSs for 15 patients in relation to the RCB scores of their residual tumors. Wilcoxon-Mann-Whitney test between RCB scores 0&1 (N=7) versus 2&3 (N=8) groups p-value=0.338. (E) Box plot of the docetaxel DSSs for 15 patients in relation to the pathological response evaluations of their residual tumors. Wilcoxon-Mann-Whitney test between pNR&pPR(N=9) versus pCR (N=6) groups p-value=0.711. (F) Box plot of the paclitaxel DSSs for 15 patients in relation to the RCB scores of their residual tumors. Wilcoxon-Mann-Whitney test between RCB scores 0&1 (N=7) versus 2&3 (N=8) groups p-value=0.197. (G) Box plot of the paclitaxel DSSs for 15 patients in relation to the pathological response evaluations of their residual tumors. Wilcoxon-Mann-Whitney test between pNR&pPR(N=9) versus pCR (N=6) groups p-value=0.595. (H) The timeline of diagnosis and treatment procedures for patient NP-5. (I) The timeline of diagnosis and treatment procedures for patient NP-2. (J) The timeline of diagnosis and treatment procedures for patient NP-4. Mammography images for each radiology check-up are shown below. Red circles indicate the tumor area. EC: epirubicin + cyclophosphamide; CR: complete response; PR: partial response; RCB: residual cancer burden; SD: stable disease; PD: progressive disease.

Table S1

ensemblid	entrezid	symbol	logFC	logCPM	LR	PValue	FDR
ENSG00000183668	5678	PSG9	7,66	2,68	101,65	6,63E-24	2,11E-20
ENSG00000163735	6374	CXCL5	7,44	5,23	59,23	1,40E-14	7,15E-12
ENSG00000169469	6699	SPRR1B	7,05	2,84	56,27	6,32E-14	2,44E-11
ENSG00000148677	27063	ANKRD1	6,88	2,81	65,30	6,44E-16	5,47E-13
ENSG00000140465	1543	CYP1A1	6,81	3,81	153,15	3,56E-35	4,53E-31
ENSG00000248779	NA	NA	-6,71	2,11	50,17	1,41E-12	3,99E-10
ENSG00000189001	374897	SBSN	6,57	5,06	105,44	9,77E-25	4,15E-21
ENSG00000155495	9947	MAGEC1	6,45	2,51	14,34	1,52E-04	1,88E-03
ENSG00000141668	147381	CBLN2	-6,28	3,27	83,69	5,78E-20	9,20E-17
ENSG00000235584	NA	NA	-6,24	3,11	71,58	2,67E-17	3,09E-14
ENSG00000183888	149563	SRARP	-5,96	3,27	85,88	1,91E-20	4,06E-17
ENSG00000110077	64231	MS4A6A	-5,85	3,65	105,45	9,71E-25	4,15E-21
ENSG00000143469	255928	SYT14	5,28	1,63	32,43	1,24E-08	8,85E-07
ENSG00000244791	101927657	LOC101927657	5,13	1,45	41,29	1,31E-10	1,88E-08
ENSG00000140297	9245	GCNT3	5,11	1,69	16,97	3,79E-05	6,22E-04
ENSG00000173335	128822	CST9	-5,00	3,15	37,79	7,90E-10	8,45E-08
ENSG00000115602	9173	IL1RL1	4,95	2,60	34,41	4,46E-09	3,84E-07
ENSG00000153294	221393	ADGRF4	4,88	3,69	49,11	2,42E-12	6,18E-10
ENSG00000169429	3576	CXCL8	4,88	8,44	61,32	4,84E-15	2,68E-12
ENSG00000204941	5673	PSG5	4,86	2,74	41,58	1,13E-10	1,69E-08

Table S1: Lists of the top 20 most differentially expressed genes as identified by differential expression analysis between TSC and WTC conditions. ensemblid: Ensembl ID, entrezid: Entrez ID, logFC: log2-fold change; logCPM: average log2-counts per million (CPM); LR: likelihood ratio statistic; FDR: false discovery rate (q-value).Results were filtered by FDR < 0.001 and sorted by the absolute value of logFC.

Table S2

NAME	SIZE	ES	NES	NOM.p.val	FDR.q.val
HALLMARK_INFLAMMATORY_RESPONSE	150	0,29	4,17	0	0
HALLMARK_TNFA_SIGNALING_VIA_NFKB	180	0,25	3,94	0	0
HALLMARK_APICAL_JUNCTION	151	0,24	3,44	0	0
HALLMARK_MITOTIC_SPINDLE	194	0,19	3,15	0	0
HALLMARK_PI3K_AKT_MTOR_SIGNALING	92	0,27	2,94	0	0
HALLMARK_COMPLEMENT	155	0,20	2,82	0	0
HALLMARK_OXIDATIVE_PHOSPHORYLATION	193	-0,17	-2,80	0	6,25E-04
HALLMARK_TGF_BETA_SIGNALING	49	0,33	2,68	0	0,00E+00
HALLMARK_IL6_JAK_STAT3_SIGNALING	69	0,26	2,57	0	4,38E-04
HALLMARK_CHOLESTEROL_HOMEOSTASIS	68	0,25	2,37	0	0,001444266
HALLMARK_MTORC1_SIGNALING	194	0,14	2,35	0	0,001638265
HALLMARK_HYPOXIA	169	0,16	2,32	0	0,002049538
HALLMARK_PROTEIN_SECRETION	92	0,20	2,27	0	0,002815215
HALLMARK_FATTY_ACID_METABOLISM	132	-0,17	-2,26	0	0,009422354
HALLMARK_ANDROGEN_RESPONSE	94	0,20	2,25	0,002040816	0,003099388
HALLMARK_EPITHELIAL_MESENCHYMAL_TRANSITION	172	0,14	2,18	0,003944773	0,004385323
HALLMARK_APOPTOSIS	142	0,16	2,17	0,001915709	0,004176918
HALLMARK_P53_PATHWAY	185	0,14	2,16	0,001976285	0,004110653
HALLMARK_KRAS_SIGNALING_UP	146	0,15	2,16	0,003976143	0,00386885
HALLMARK_ALLOGRAFT_REJECTION	157	0,14	2,11	0,001972387	0,004803267

Table S2: Lists of the significantly affected pathways as identified by GSEA (MSigDB Hallmarks gene-set collection), between TSC and WTC conditions.
SIZE: # of genes in gene-set; ES: enrichment score; NES: normalized enrichment score; NOM.p.val: nominal p-value; FDR.q.val: false discovery rate. Results were filtered by FDR < 0.01 and sorted by the absolute value of NES.

Table S3

NAME	SIZE	ES	NES	NOM.p.val	FDR.q.val
HALLMARK_E2F_TARGETS	194	-0,58	-2,78	0	0
HALLMARK_G2M_CHECKPOINT	193	-0,56	-2,74	0	0
HALLMARK_ESTROGEN_RESPONSE_LATE	188	-0,54	-2,67	0	0
HALLMARK_ESTROGEN_RESPONSE_EARLY	194	-0,51	-2,52	0	0
HALLMARK_TNFA_SIGNALING_VIA_NFKB	186	-0,38	-1,84	0	0,003220213
HALLMARK_INTERFERON_ALPHA_RESPONSE	93	0,44	1,83	0	0,005141773
HALLMARK_MITOTIC_SPINDLE	193	-0,36	-1,78	0	0,003364432
HALLMARK_UV_RESPONSE_UP	139	-0,36	-1,68	0	0,008114333
HALLMARK_APOPTOSIS	144	-0,35	-1,65	0	0,009985229

Table S3: Lists of the significantly affected pathways as identified by GSEA (MSigDB Hallmarks gene-set collections), between 4OHT treated and untreated WTCs. SIZE: # of genes in gene-set; ES: enrichment score; NES: normalized enrichment score; NOM.p.val: nominal p-value; FDR.q.val: false discovery rate. Results were filtered by FDR < 0.01 and sorted by the absolute value of NES.

Table S4

NAME	SIZE	ES	NES	NOM.p.val	FDR.q.val
HALLMARK_E2F_TARGETS	194	-0,68	-3,50	0	0
HALLMARK_G2M_CHECKPOINT	193	-0,63	-3,26	0	0
HALLMARK_ESTROGEN_RESPONSE_LATE	188	-0,50	-2,59	0	0
HALLMARK_HYPOXIA	179	0,45	2,27	0	0
HALLMARK_MYC_TARGETS_V2	56	-0,50	-2,15	0	0,00E+00
HALLMARK_PROTEIN_SECRETION	94	0,46	2,12	0	0,00E+00
HALLMARK_SPERMATOGENESIS	66	-0,46	-1,99	0	8,94E-04
HALLMARK_ESTROGEN_RESPONSE_EARLY	194	-0,37	-1,94	0	8,82E-04
HALLMARK_DNA_REPAIR	143	-0,37	-1,86	0	1,43E-03
HALLMARK_UV_RESPONSE_UP	139	-0,37	-1,82	0	0,001643422
HALLMARK_MITOTIC_SPINDLE	193	-0,35	-1,81	0	0,00146082
HALLMARK_MYC_TARGETS_V1	197	-0,31	-1,65	0	0,007998779

Table S4: Lists of the significantly affected pathways as identified by GSEA (MSigDB Hallmarks gene-set collections), between EDF treated and untreated WTCs. SIZE: # of genes in gene-set; ES: enrichment score; NES: normalized enrichment score; NOM.p.val: nominal p-value; FDR.q.val: false discovery rate. Results were filtered by FDR < 0.01 and sorted by the absolute value of NES.

Table S5

NAME	SIZE	ES	NES	NOM.p.val	FDR.q.val
PID_PLK1_PATHWAY	43	-0,65	-2,46	0	0
REACTOME_CELL_CYCLE_MITOTIC	475	-0,44	-2,40	0	0
REACTOME_RESOLUTION_OF_SISTER_CHROMATID_COHESION	108	-0,53	-2,37	0	0
KEGG_CELL_CYCLE	116	-0,52	-2,36	0	0
PID_ATR_PATHWAY	36	-0,64	-2,35	0	0
REACTOME_MITOTIC_PROMETAPHASE	180	-0,48	-2,31	0	0
REACTOME_CELL_CYCLE_CHECKPOINTS	249	-0,45	-2,31	0	1,94E-04
REACTOME_RHO_GTPASES_ACTIVATE_FORMINS	121	-0,51	-2,31	0	1,70E-04
PID_FOXM1_PATHWAY	37	-0,63	-2,28	0	3,11E-04
REACTOME_DNA_STRAND_ELONGATION	32	-0,63	-2,21	0	0,001126415
REACTOME_MITOTIC_G1_PHASE_AND_G1_S_TRANSITION	142	-0,47	-2,20	0	0,001286722
REACTOME_MITOTIC_METAPHASE_AND_ANAPHASE	210	-0,43	-2,16	0	0,002008966
REACTOME_KINESINS	43	-0,57	-2,12	0	0,003599431
KEGG_DNA_REPLICATION	34	-0,61	-2,12	0	0,003546341
REACTOME_ACTIVATION_OF_ATR_IN_RESPONSE_TO_REPLICATION_STRESS	35	-0,59	-2,12	0	0,003309918
REACTOME_G1_S_SPECIFIC_TRANSCRIPTION	28	-0,62	-2,10	0	0,004162554
REACTOME_MITOTIC_SPINDLE_CHECKPOINT	101	-0,47	-2,09	0	0,004669631

Table S5: Lists of the significantly affected pathways as identified by GSEA (MSigDB Canonical pathways gene-set collections), between 4OHT treated and untreated WTCs. SIZE: # of genes in gene-set; ES: enrichment score; NES: normalized enrichment score; NOM.p.val: nominal p-value; FDR.q.val: false discovery rate. Results were filtered by FDR < 0.005 and sorted by the absolute value of NES.

Table S6

NAME	SIZE	ES	NES	NOM.p.val	FDR.q.val
REACTOME_CELL_CYCLE_MITOTIC	475	-0,49	-2,87	0	0
REACTOME_MITOTIC_PROMETAPHASE	180	-0,55	-2,86	0	0
REACTOME_RESOLUTION_OF_SISTER_CHROMATID_COHESION	108	-0,60	-2,85	0	0
REACTOME_CELL_CYCLE_CHECKPOINTS	249	-0,53	-2,84	0	0
REACTOME_RHO_GTPASES_ACTIVATE_FORMINS	121	-0,59	-2,81	0	0
REACTOME_DNA_REPLICATION	119	-0,57	-2,72	0	0
REACTOME_MITOTIC_SPINDLE_CHECKPOINT	101	-0,58	-2,72	0	0
REACTOME_DNA_STRAND_ELONGATION	32	-0,74	-2,71	0	0
PID_PLK1_PATHWAY	43	-0,68	-2,68	0	0
KEGG_CELL_CYCLE	116	-0,56	-2,68	0	0
REACTOME_CHROMOSOME_MAINTENANCE	75	-0,60	-2,67	0	0
PID_AURORA_B_PATHWAY	35	-0,70	-2,62	0	0
KEGG_DNA_REPLICATION	34	-0,71	-2,62	0	0
REACTOME_SEPARATION_OF_SISTER_CHROMATIDS	166	-0,52	-2,61	0	0
REACTOME_ACTIVATION_OF_THE_PRE_REPLICATIVE_COMPLEX	31	-0,73	-2,60	0	0
REACTOME_MITOTIC_METAPHASE_AND_ANAPHASE	210	-0,48	-2,56	0	0
REACTOME_ACTIVATION_OF_ATR_IN_RESPONSE_TO_REPLICATION_STRESS	35	-0,67	-2,55	0	0
REACTOME_MITOTIC_G1_PHASE_AND_G1_S_TRANSITION	142	-0,50	-2,54	0	0
REACTOME_G2_M_CHECKPOINTS	135	-0,51	-2,51	0	0
REACTOME_M_PHASE	337	-0,45	-2,51	0	0
REACTOME_DNA_REPLICATION_PRE_INITIATION	78	-0,54	-2,48	0	0
PID_FOXM1_PATHWAY	37	-0,65	-2,47	0	0
REACTOME_EUKARYOTIC_TRANSLATION_ELONGATION	87	-0,53	-2,44	0	0
REACTOME_DEPOSITION_OF_NEW_CENPA_CONTAINING_NUCLEOSOMES_AT_THE_CENTROMERE	36	-0,64	-2,43	0	0
PID_HIF2PATHWAY	27	0,68	2,38	0	0
PID_ATR_PATHWAY	36	-0,62	-2,37	0	0
REACTOME_S_PHASE	153	-0,48	-2,37	0	0
KEGG_RIBOSOME	82	-0,52	-2,36	0	0
REACTOME_APC_C_MEDIATED_DEGRADATION_OF_CELL_CYCLE_PROTEINS	80	-0,52	-2,35	0	0

REACTOME_HOMOLOGY_DIRECTED_REPAIR	104	-0,50	-2,33	0	0,00E+00
REACTOME_CYCLIN_A_B1_B2_ASSOCIATED_EVENTS_DURING_G2_M_TRANSITION	24	-0,67	-2,29	0	3,38E-05
REACTOME_TELOMERE_MAINTENANCE	52	-0,56	-2,29	0	3,27E-05
REACTOME_KINESINS	43	-0,58	-2,28	0	3,17E-05
REACTOME_G1_S_SPECIFIC_TRANSCRIPTION	28	-0,64	-2,28	0	3,07E-05
PID_HIF1_TFPATHWAY	58	0,55	2,26	0	0,00E+00
REACTOME_TELOMERE_C_STRAND_LAGGING_STRAND_SYNTHESIS	27	-0,64	-2,26	0	8,65E-05
BIOCARTA_G2_PATHWAY	23	-0,65	-2,25	0	1,13E-04
REACTOME_RHO_GTPASE_EFFECTORS	243	-0,42	-2,25	0	1,40E-04
REACTOME_PROCESSING_OF_DNA_DOUBLE_STRAND_BREAK_ENDS	71	-0,51	-2,24	0	1,36E-04
REACTOME_HDR_THROUGH_HOMOLOGOUS_RECOMBINATION_HRR	59	-0,52	-2,23	0	1,33E-04
REACTOME_BASE_EXCISION_REPAIR	55	-0,53	-2,23	0	2,08E-04
PID_AURORA_A_PATHWAY	29	-0,62	-2,21	0	2,53E-04
KEGG_PARKINSONS_DISEASE	112	-0,46	-2,19	0	3,71E-04
REACTOME_PCNA_DEPENDENT_LONG_PATCH_BASE_EXCISION_REPAIR	20	-0,68	-2,18	0	4,11E-04
REACTOME_RESOLUTION_OF_ABASIC_SITES_AP_SITES	37	-0,57	-2,18	0	4,24E-04
REACTOME_MITOTIC_G2_G2_M_PHASES	178	-0,43	-2,18	0	4,15E-04
REACTOME_DNA_DOUBLE_STRAND_BREAK_REPAIR	129	-0,45	-2,18	0	4,05E-04
REACTOME_EXTENSION_OF_TELOMERES	40	-0,55	-2,18	0	4,19E-04
KEGG_BASE_EXCISION_REPAIR	31	-0,59	-2,15	0	7,33E-04
REACTOME_ASSEMBLY_OF_THE_PRE_REPLICATIVE_COMPLEX	63	-0,50	-2,14	0	8,02E-04
KEGG_OXIDATIVE_PHOSPHORYLATION	115	-0,44	-2,13	0	8,48E-04
REACTOME_APC_CDC20_MEDIATED_DEGRADATION_OF_NEK2A	24	-0,62	-2,13	0	8,31E-04
REACTOME_RECRUITMENT_OF_NUMA_TO_MITOTIC_CENTROSOMES	81	-0,47	-2,12	0	0,001069
REACTOME_G2_M_DNA_DAMAGE_CHECKPOINT	67	-0,49	-2,11	0	0,001184
REACTOME_THE_ROLE_OF_GTSE1_IN_G2_M_PROGRESSION_AFTER_G2_CHECKPOINT	64	-0,49	-2,10	0	0,00122
REACTOME_RESPONSE_OF_EIF2AK4_GCN2_TO_AMINO_ACID_DEFICIENCY	96	-0,45	-2,10	0	0,001217
REACTOME_RESOLUTION_OF_AP_SITES_VIA_THE_MULTIPLE_NUCLEOTIDE_PATCH_REPLACEMENT_PATHWAY	24	-0,61	-2,10	0	0,001194
REACTOME_CONDENSATION_OF_PROPHASE_CHROMOSOMES	27	-0,60	-2,10	0	0,001173
REACTOME_RESPIRATORY_ELECTRON_TRANSPORT_ATP_SYNTHESIS_BY_CHEMIOSMOTIC_COUPLING_AND_HEAT_PRODUCTION_BY_UNCOUPLING_PROTEINS	114	-0,43	-2,10	0	0,001204

REACTOME_PROCESSIVE_SYNTHESIS_ON_THE_LAGGING_STRAND	15	-0,69	-2,09	0,0019960 1	0,001306
REACTOME_RESOLUTION_OF_D_LOOP_STRUCTURES	28	-0,58	-2,07	0	0,001492
REACTOME_G0_AND_EARLY_G1	26	-0,60	-2,07	0	0,001484
REACTOME_SWITCHING_OF_ORIGINS_TO_A_POST_REPLICATIVE_STATE	84	-0,46	-2,07	0	0,00146
REACTOME_LAGGING_STRAND_SYNTHESIS	20	-0,65	-2,06	0	0,00152
REACTOME_RESPIRATORY_ELECTRON_TRANSPORT	93	-0,44	-2,05	0	0,001853
REACTOME_HOMOLOGOUS_DNA_PAIRING_AND_STRAND_EXCHANGE	39	-0,54	-2,04	0,0021551 7	0,001984
REACTOME_AURKA_ACTIVATION_BY_TPX2	68	-0,48	-2,04	0	0,002062
PID_FANCONI_PATHWAY	41	-0,52	-2,01	0	0,002702
REACTOME_COMPLEX_I_BIOGENESIS	50	-0,49	-2,01	0	0,002932
BIOCARTA_CELLCYCLE_PATHWAY	22	-0,60	-2,00	0	0,003113
REACTOME_APC_C_CDC20_MEDIATED_DEGRADATION_OF_CYCLIN_B	22	-0,60	-2,00	0	0,00329
REACTOME_RESOLUTION_OF_D_LOOP_STRUCTURES	23	-0,61	-1,99	0	0,003358
REACTOME_ORC1_REMOVAL_FROM_CHROMATIN	66	-0,46	-1,99	0	0,003325
BIOCARTA_MCM_PATHWAY	18	-0,64	-1,98	0	0,003772
REACTOME_PHOSPHORYLATION_OF_THE_APC_C	18	-0,63	-1,98	0	0,003862
REACTOME_POLYMERASE_SWITCHING_ON_THE_C_STRAND_OF_THE_TELOMERE	17	-0,64	-1,97	0,0021322	0,004208
REACTOME_RECRUITMENT_OF_MITOTIC_CENTROSOME_PROTEINS_AND_COMPLEXES	75	-0,44	-1,96	0	0,004395
PID_BARD1_PATHWAY	28	-0,55	-1,96	0	0,004954

Table S6: Lists of the significantly affected pathways as identified by GSEA (MSigDB Canonical pathways gene-set collections), between EDF treated and untreated WTCs. SIZE: # of genes in gene-set; ES: enrichment score; NES: normalized enrichment score; NOM.p.val: nominal p-value; FDR.q.val: false discovery rate. Results were filtered by FDR < 0.005 and sorted by the absolute value of NES.

List S1 – Patient tumor information

Patient ID	Age	ER (%)	PR (%)	HER2_IHC score	HER2_SISH	HER2_status	Ki67 (%)	Tumor_size (mm)	Elston_grade	T_stage	N_stage	Metastasis positive node number	Total examined node number	Subtype	Neoadjuvant therapy received	Primary tumor
**IHC-1**	87	0	0	0	-	neg	95	47	3	2	1	2	26	TNBC	No	Yes
**IHC-2**	72	100	50	2	neg	neg	65	27	3	2	0	0	3	LB/H2-	No	Yes
**IHC-3**	86	100	1	0	-	neg	50	27	3	2	0	0	11	LB/H2-	No	Yes
**IHC-4**	47	0	0	1	-	neg	81	34	3	2	0	0	5	TNBC	No	Yes
**IHC-5**	85	99	99	0	-	neg	22	47	2	2	2	4	15	LB/H2-	No	Yes
**IHC-6**	63	99	99	0	-	neg	24	19	2	1	0	0	2	LB/H2-	No	Yes
**IHC-7**	78	95	95	0-1	-	neg	36	18	3	1	0	0	2	LB/H2-	No	Yes
**IHC-8**	36	90	80	1-2	neg	neg	18	30	2	2	1	1	1	LA	No	Yes
**IHC-9**	53	90	55	1-2	neg	neg	50	23	3	2	0	0	4	LB/H2-	No	Yes
**IHC-10**	N/A	95	75	0-1	-	neg	30	115	3	3	2	7	12	LB/H2-	Yes	Yes
**IHC-11**	73	95	15	0-1	-	neg	26	14	2	1	0	0	1	LB/H2-	No	Yes
**IHC-12**	69	0	0	0	-	neg	90	170	3	3	2	5	9	TNBC	Yes	Yes
**IF-1**	83	99	90	0	-	neg	68	32	2	2	1	3	12	LB/H2-	No	Yes
**IF-2**	35	60	20	2	neg	neg	70	12	3	1	0	0	2	LB/H2-	No	Yes
**IF-3**	37	2	1	0	-	neg	90	17	3	1	1	1	1	TNBC	No	Yes
**IF-4**	51	99	80	1	-	neg	19	55	1	3	2	4	16	LA	No	Yes
**IF-5**	71	100	5	0	-	neg	21	15	2	1	0	0	2	LB/H2-	No	Yes
**FACS-1**	66	95	<5	1	-	neg	32	12	3	1	1	2	8	LB/H2-	No	Yes
**FACS-2**	67	100	50	2	neg	neg	25	25	2	2	1	1	2	LB/H2-	No	Yes
**FACS-3**	46	98	100	1	neg	neg	12	34	2	2	1	2	4	LA	No	Yes
**FACS-4**	77	99	70	1	-	neg	75	40	3	2	1	2	12	LB/H2-	No	Yes
**FACS-5**	73	70	80	0	-	neg	16	15	2	1	0	0	3	LA	No	Yes
**FACS-6**	82	0	0	0	-	neg	72	67	3	3	2	9	15	TNBC	No	Yes
**FACS-7**	67	100	95	0	-	neg	37	17	2	1	1	1	1	LB/H2-	No	Yes
**FACS-8**	72	99	70	2	neg	neg	67	22	3	2	0	0	1	LB/H2-	No	Yes
**FACS-9**	65	100	65	1-2	neg	neg	15	14	1	1	0	0	2	LA	No	Yes
**FACS-10**	76	100	90	1+	-	neg	27	15	2	1	0	0	1	LB/H2-	No	Yes
**FACS-11**	83	99	90	0	-	neg	68	32	2	2	1	3	12	LB/H2-	No	Yes

**FACS-12**	79	0	0	0	-	neg	98	37	3	2	N/A	>1	N/A	TNBC	No	Yes
**FACS-13**	84	20	0	0	-	neg	90	34	3	2	0	0	2	LB/H2-	No	Yes
**FACS-14**	92	95	85	1-2	neg	neg	44	35	2	2	1	1	12	LB/H2-	No	Yes
**RI-1**	65	100	95	1	-	neg	20	21	2	2	1	2	2	LB/H2-	No	Yes
**RI-2**	57	100	100	3	pos	pos	22	65	3	3	0	0	2	LB/H2+	Yes	No
**RI-3**	42	95	100	1	-	neg	24	24	2	2	2	4	4	LB/H2-	No	Yes
**DNA-1**	64	0	0	3	pos	pos	60	20	3	1	0	0	3	H2+	No	Yes
**DNA-2**	43	20	20	3	-	pos	42	82	3	3	2	9	19	LB/H2+	No	Yes
**DNA-3**	62	99	70	0	-	neg	40	18	2	1	2	5	19	LB/H2-	No	Yes
**DNA-4**	84	95	90	0	-	neg	40	22	3	2	0	0	4	LB/H2-	No	Yes
**DNA-5**	55	95	99	0	-	neg	23	16	2	1	1	1	10	LB/H2-	No	Yes
**RNA-1**	47	20	0	0	-	neg	75	43	3	2	0	0	9	LB/H2-	Yes	Yes
**RNA-2**	71	100	90	2	neg	neg	27	16	3	1	0	0	2	LB/H2-	No	Yes
**RNA-3**	87	100	5	0-1	-	neg	70	18	3	1	2	4	14	LB/H2-	No	Yes
**RNA-4**	65	0	0	0	-	neg	70	16	3	1	0	0	2	TNBC	No	Yes
**RNA-5**	49	95	70	1	-	neg	5	18	1	1	0	0	3	LA	No	Yes
**RNA-6**	47	95	95	1	-	neg	24	20	2	1	0	0	1	LB/H2-	No	Yes
**RNA-7**	60	95	5	1	-	neg	20	23	2	2	0	0	5	LB/H2-	No	Yes
**RNA-8**	58	100	20	2	neg	neg	35	25	3	2	2	7	24	LB/H2-	No	Yes
**RNA-9**	25	100	25	2	pos	pos	20	14	2	1	1	1	2	LB/H2+	No	Yes
**RNA-10**	86	100	95	2	neg	neg	31	42	2	2	N/A	N/A	N/A	LB/H2-	No	Yes
**RNA-11**	44	100	100	3	pos	pos	80	38	3	2	1	1	2	LB/H2+	No	Yes
**RNA-12**	47	90	95	1	-	neg	40	19	2	1	0	0	2	LB/H2-	No	Yes
**RNA-13**	71	99	0	1	-	neg	40	45	N/A	2	0	0	2	LB/H2-	No	Yes
**RNA-14**	47	70	85	1	-	neg	27	13	2	1	1	2	13	LB/H2-	No	Yes
**RNA-15**	40	-	-	-	-	-	-	-	-	-	-	-	-	RADIAL SCAR	-	-
**RNA-16**	62	0	0	0	-	neg	65	35	3	2	0	0	8	TNBC	No	Yes
**DP-1**	48	95	40	1	-	neg	3	11	1	1	1	1	12	LA	Yes	Yes
**DP-2**	83	99	0	0	-	neg	5	60	2	3	2	6	19	LB/H2-	Yes	Yes
**DP-3**	52	90	0	3	neg	neg	7	32	2	2	0	0	1	LB/H2-	Yes	Yes

**DP-4**	67	100	90	1	-	neg	12	3	2	1	0	0	14	LA	No	Yes
**DP-5**	50	90	95	1	-	neg	15	16	1	1	0	0	4	LA	No	Yes
**DP-6**	71	99	99	0	-	neg	15	37	2	2	0	0	2	LA	No	Yes
**DP-7**	73	99	90	0	-	neg	15	12	1	1	0	0	1 (ITC+)	LA	No	Yes
**DP-8**	45	99	95	0	-	neg	18	15	2	1	0	0	3	LA	No	Yes
**DP-9**	79	99	99	2	neg	neg	19	80	2	3	3	10	16	LA	Yes	Yes
**DP-10**	60	99	25	0	-	neg	19	40	2	2	2	4	12	LA	No	Yes
**DP-11**	84	99	100	0	-	neg	22	33	2	2	2	5	20	LB/H2-	No	Yes
**DP-12**	54	99	99	0	-	neg	23	17	2	1	0	0	2	LB/H2-	No	Yes
**DP-13**	59	99	80	0	-	neg	26	26	3	2	0	0	2 (ITC+)	LB/H2-	No	Yes
**DP-14**	83	70	0	3	-	pos	27	70	3	3	0	0	2	LB/H2+	No	Yes
**DP-15**	73	100	100	0	-	neg	28	16	2	1	0	0	1 (ITC+)	LB/H2-	No	Yes
**DP-16**	60	99	90	1	-	neg	35	24	2	2	0	0	1	LB/H2-	No	Yes
**DP-17**	44	99	99	0	-	neg	35	37	3	2	1	1	13	LB/H2-	No	Yes
**DP-18**	62	0	0	3	-	pos	40	17	3	1	0	0	1	H2+	No	Yes
**DP-19**	70	100	100	0	-	neg	36	14	2	1	0	0	1	LB/H2-	No	Yes
**DP-20**	84	90	40	3	-	pos	37	33	3	2	1	1	14	LB/H2+	No	Yes
**DP-21**	67	100	40	2	neg	neg	38	22	2	2	1	1	16	LB/H2-	No	Yes
**DP-22**	72	99	85	1	-	neg	39	22	2	2	0	0	1	LB/H2-	No	Yes
**DP-23**	65	100	100	0	-	neg	40	9	2	1	0	0	4 (ITC+)	LB/H2-	No	Yes
**DP-24**	79	100	70	0	-	neg	40	21	3	2	1	1	23	LB/H2-	No	Yes
**DP-25**	82	0	0	0	-	neg	41	54	3	3	1	3	15	TNBC	No	Yes
**DP-26**	56	90	90	1	-	neg	44	20	3	1	1	1	2	LB/H2-	No	Yes
**DP-27**	79	99	93	1	-	neg	46	40	3	2	0	0	3 (ITC+)	LB/H2-	No	Yes
**DP-28**	51	95	95	0	-	neg	46	21	2	2	0	0	3 (ITC+)	LB/H2-	No	Yes
**DP-29**	87	99	30	0	-	neg	46	35	3	2	NA	NA	NA	LB/H2-	No	Yes
**DP-30**	52	100	<1	1	-	neg	48	20	3	1	0	0	2 (ITC+)	LB/H2-	No	Yes
**DP-31**	85	80	1	2	neg	neg	50	31	3	2	0	0	3	LB/H2-	Yes	Yes
**DP-32**	69	99	1	1	-	neg	50	22	3	2	0	0	2	LB/H2-	No	Yes
**DP-33**	73	0	0	3	-	pos	50	21	3	2	0	0	2	H2+	No	Yes

**DP-34**	60	60	0	3	-	pos	60	21	3	2	0	0	4	LB/H2+	No	Yes
**DP-35**	51	<5	0	3	pos	pos	65	13	3	1	0	0	1	LB/H2+	No	Yes
**DP-36**	84	0	5	0	-	neg	70	16	3	1	0	0	2 (ITC+)	TNBC	No	Yes
**DP-37**	88	0	0	0	-	neg	70	28	3	2	0	0	2	TNBC	No	Yes
**DP-38**	58	0	0	0	-	neg	70	125	3	3	0	0	11	TNBC	No	Yes
**DP-39**	49	0	0	0	-	neg	70	33	3	2	1	1	10	TNBC	Yes	Yes
**DP-40**	94	0	0	2	neg	neg	70	35	3	2	NA	NA	NA	TNBC	No	Yes
**DP-41**	97	0	0	0	-	neg	71	30	3	2	NA	NA	NA	TNBC	No	Yes
**DP-42**	88	0	0	0	-	neg	80	24	3	2	0	0	8	TNBC	No	Yes
**DP-43**	77	99	80	2	neg	neg	90	38	3	2	0	0	1	LB/H2-	No	Yes
**DP-44**	77	0	0	0	-	neg	90	13	3	1	0	0	2	TNBC	No	Yes
**DP-45**	75	0	0	0	-	neg	92	43	3	2	1	1	13	TNBC	No	Yes

List S1 – Patient tumor information

Patient ID	Age	ER (%)	PR (%)	HER2_IHC score	Her2_SISH	Her2_status	Ki67 (%)	Elston_grade	Subtype	Node metastasis before treatment	Radiological tumor size before neoadjuvant (mm)	Radiological tumor size after EC (mm)	RECIST Criteria after EC	Pathological response after all treatments	RCB score	Residual tumor grade	Residual tumor size (mm)	Residual tumor ER(%)	Residual tumor PR(%)	Residual tumor Ki67(%)	Residual tumor HER2 (IHC score)	Residual tumor HER2 (SISH)	Residual tumor HER2 status	Residual tumor lymph node status	comment
**NP-1**	36	95	90	2+	neg	neg	25	2	LB/H2-	yes	48	48	SD	pNR	3	2	37	99	0	7	1 to 2+	SISH neg	neg	5/12	-
**NP-2**	74	0	0	2+	neg	neg	29	2	TNBC	N/A	28	20	SD	pPR	2	2	31	0	0	5	2+	SISH neg	neg	0/2	-
**NP-3**	35	95	20	0	-	neg	80	3	LB/H2-	yes	72	60	SD	pPR	3	3	17	90	0	83	0	N/A	neg	5/12	-
**NP-4**	47	<1	0	3+	-	pos	65	3	H2+	N/A	16	0	CR	pCR	0	-	-	-	-	-	-	-	-	0/1	-
**NP-5**	56	<1	0	0	-	neg	80	3	TNBC	N/A	20	0	CR	pCR	0	-	-	-	-	-	-	-	-	0/1	-
**NP-6**	51	80	65	3+	-	pos	53	3	LB/H2+	yes	30	30	SD	pCR	0	-	-	-	-	-	-	-	-	0/21	DCIS remaining
**NP-7**	49	70	5	3+	-	pos	30	2	LB/H2+	yes	35	12	PR	pCR	0	-	-	-	-	-	-	-	-	0/10	-
**NP-8**	44	2	2	3+	-	pos	57	3	H2+	yes	21	17	SD	pPR	2	3	29	0	0	63	3+	N/A	pos	2/21	-
**NP-9**	35	0	0	0	-	neg	55	2	TNBC	yes	22	15	PR	pPR	1	-	-	-	-	-	-	-	-	1/10	Minimal DCIS remaining
**NP-10**	46	0	0	1+	-	neg	91	3	TNBC	yes	27	0	CR	pCR	0	-	-	-	-	-	-	-	-	-	-
**NP-11**	33	0	0	1+	-	neg	75	3	TNBC	no	34	14	PR	pPR	2	3	18	0	0	53	1+	N/A	neg	1/2	-
**NP-12**	56	100	80	2+	neg	neg	38	3	LB/H2-	yes	30	23	SD	pPR	3	2	17	85	50	8	2+	pos	pos	2/21	-
**NP-13**	57	95	95	0-1+	-	neg	40	2	LB/H2-	no	33	20	PR	pPR	3	2	45	80	0	1	0	N/A	neg	2/4	-
**NP-14**	64	0	0	0	-	neg	70	3	TNBC	yes	29	25	SD	pPR	2	2	28	0	2	27	0	N/A	neg	1/9	-
**NP-15**	49	0	5	0	-	neg	80	3	TNBC	N/A	15	0	CR	pCR	0	-	-	-	-	-	-	-	-	0/1	-

List S1 – Patient tumor information

Patient ID	EC start	EC stop	Detailed treatment information - EC (epirubicin + cyclophosphamide)	Doc/Pac start	Doc/Pac stop	Detailed treatment information - docetaxel/paclitaxel (Doc/Pac)	Tras/Per start	Tras/Per stop	Detailed treatment information - trastuzumab/pertuzumab (Tras/Per)
**NP-1**	2020-09-29	2020-11-10	ddEC x 4: Epirubicin total dose 772 mg (465 mg/m2), Cyclophosphamide total dose 7470 mg (4500mg/m2). dd=dose dense and escalated.	2020-12-01	2021-01-15	ddDocetaxel 75 mg /m2 x 4, q2w, total dose 498 mg (300 mg/m2). dd=dose dense and ecsalated. q2w=given every 2 weeks.	-	-	NA
**NP-2**	2020-10-07	2020-11-18	EC75 x 3 (q3w). Epirubicin total dose 369 mg (225 mg/m2), Cyclophosphamide total dose 2950 mg (1800 mg /m2). q3w=given every 3 weeks.	2020-12-09	2020-12-30	Paclitaxel x 4 (out of 9 planned cycles), stopped early due to side effects. Total dose 525 mg (320 mg/m2) .	-	-	NA
**NP-3**	2020-10-23	2020-12-04	ddEC x 4: Epirubicin total dose 748 mg (465 mg/m2), Cyclophosphamide total dose 7245 mg (4500mg/m2).	2020-12-29	2021-01-12	ddDocetaxel 75 mg /m2 x 2, q2w, total dose 241 mg (150 mg/m2). Treatment stopped early after 2 out of 4 planned cycles due to side effects. q2w=given every 2 weeks.	-	-	NA
**NP-4**	2020-10-09	2020-11-20	dEC90 x 4: Epirubicin total dose 558 mg (360 mg/m2), Cyclophosphamide total dose 3720 mg (2400 mg/m2). d=dose dense.	2020-12-11	2021-01-26	Docetaxel 100 mg/m2 x 1 and docetaxel 90 mg/m2 x 2, q3w, total dose 434 mg (280 mg/m2). Dose reduction due to side effects. q3w=given every 3 weeks.	2020-12-11	2021-01-26	Trastuzumab iv 8 mg/kg x 1 and 6 mg/kg x 3. Total dose 1404 mg. Pertuzumab iv 840 mg x 1 and 420 mg x 3. Total dose=2100 mg.
**NP-5**	2020-10-12	2020-11-24	ddEC x 4: Epirubicin total dose 700 mg (405 mg/m2), Cyclophosphamide total dose 5709 mg (3300 mg/m2).	2020-12-21	2020-12-29	Paclitaxel x 1 (out of 9 planned cycles) 80 mg/m2, total dose 138 mg. Treatment stopped due to diverticulitis and perforation.	-	-	NA
**NP-6**	2020-11-20	2021-01-04	ddEC x 4: Epirubicin total dose 615 mg (375 mg/m2), Cyclophosphamide total dose 4428 mg (2700 mg/m2).	2021-01-25	2021-03-08	Docetaxel 100 mg/m2 x 1, Doc 75 mg/m2 x 2, total dose 410 mg (250 mg/m2). Dose reduction due to side effects.	2021-01-25	2021-03-08	Trastuzumab iv 8 mg/kg x 1 and 6 mg/kg x 3 . Total dose 1586 mg. Pertuzumab iv 840 mg x 1 and 420 mg x 3. Total dose=2100 mg.
**NP-7**	2020-12-03	2021-01-13	ddEC x 4: Epirubicin total dose 910 mg (420 mg/m2), Cyclophosphamide total dose 7803 mg (3600 mg/m2).	2021-02-03	2021-03-10	Docetaxel 100 mg/m2 x 1, and then changed to paclitaxel 80 mg/m2 x 3 (out of 6 planned cycles). Stopped early due to side effects. Total dose docetaxel 210 mg (100 mg/m2), paclitaxel 511 mg (240 mg/m2).	2021-02-03	2021-03-17	Trastuzumab iv 8 mg/kg x 1 and 6 mg/kg x 2 . Total dose 1940 mg. Pertuzumab iv 840 mg x 1 and 420 mg x 2. Total dose=1680 mg.
**NP-8**	2020-12-22	2021-02-04	ddEC x 4: Epirubicin total dose 841 mg (465 mg/m2), Cyclophosphamide total dose 8145 mg (4500 mg/m2).	2021-02-25	2021-04-22	Docetaxel 100 mg/m2 x 1, and then changed to paclitaxel 80% of 80 mg/m2 x 6 due to side effects. Total dose docetaxel 161 mg (100 mg/m2), paclitaxel 695 mg (384 mg/m2).	2021-02-25	2021-04-29	Trastuzumab iv 8 mg/kg x 1 and 6 mg/kg x 3 . Total dose 1710 mg. Pertuzumab iv 840 mg x 1 and 420 mg x 3. Total dose=2100 mg.
**NP-9**	2020-12-29	2021-02-09	ddEC x 4: Epirubicin total dose 685 mg (390 mg/m2), Cyclophosphamide total dose 5268mg (3000 mg/m2).	2021-03-02	2021-03-30	ddDocetaxel q2w x 3 (out of 4 planned cycles), total dose 441 mg (245 mg/m2), stopped early due to side effects. q2w=given every 2 weeks.	-	-	NA

**NP-10**	2020-12-17	2021-02-02	ddEC x 4: Epirubicin total dose 855 mg (450 mg/m2), Cyclophosphamide total dose 7980 mg (4200 mg/m2).	2021-02-26	2021-04-23	ddDocetaxel x 1, changed to paclitaxel x 5 (out of 6 planned cycles) due to side effects. Total dose docetaxel 142 mg (75 mg/m2) and paclitaxel 730 mg (384 mg/m2).	-	-	NA
**NP-11**	2020-12-22	2021-02-11	ddEC x 4: Epirubicin total dose 745 mg (405 mg/m2), Cyclophosphamide total dose 6072 mg (3300 mg/m2).	2021-03-04	2021-04-15	ddDocetaxel x 4. Total dose docetaxel 570 mg (310 mg/m2).	-	-	NA
**NP-12**	2021-02-04	2021-03-18	dEC90 x 4: Epirubicin total dose 623 mg (360 mg/m2), Cyclophosphamide total dose 4152mg (2400 mg/m2).	2021-04-08	2021-07-01	Paclitaxel x 12 (dose reduction course 7-12). Total dose 1536 mg (888 mg/m2).	-	-	NA
**NP-13**	2021-02-09	2021-03-23	ddEC x 4: Epirubicin total dose 717 mg (450 mg/m2), Cyclophosphamide total dose 6696 mg (4200 mg/m2).	2021-04-20	2021-04-30	ddDocetaxel x 1, stopped early due to side effects. Total dose docetaxel 120 mg (75 mg/m2).	-	-	NA
**NP-14**	2021-02-05	2021-03-19	ddEC x 4: Epirubicin total dose 735 mg (420 mg/m2), Cyclophosphamide total dose 6300 mg (3600 mg/m2).	2021-04-09	2021-05-21	ddDocetaxel x 4. Total dose docetaxel 525 mg (300 mg/m2).	-	-	NA
**NP-15**	2021-04-13	2021-05-26	ddEC x 4: Epirubicin total dose 756 mg (420 mg/m2), Cyclophosphamide total dose 6480 mg (3600 mg /m2).	2021-06-16	2021-07-28	ddDocetaxel x 4. Total dose docetaxel 566 mg (320 mg/m2).	-	-	NA

List S1 – Patient tumor information

**Surrogate subtype St Gallen 2013 - Goldhirsch et al Annals of Oncology 24:2206-2223, 2013**	
**Intrinsic surrogate subtype**	**Clinico-pathologic surrogate definition**
**Luminal A-like**	ER positive (≥1%) and PR positive (≥20%)
**LA**	*and*
	HER2 negative
	and
	Ki67 low (<20%)
**Luminal B-like (HER2 negative)**	ER positive (≥1%)
**LB/H2-**	HER2 negative
	and at least one of:
	Ki67 high (≥20%)
	PR negative or low (<20%)
**Luminal B-like (HER2 positive)**	ER positive (≥1%)
**LB/H2+**	HER2 over-expressed or amplified
	any Ki67/PR
**HER2 positive (non-luminal)**	HER2 over-expressed or amplified
**H2+**	ER and PR absent (<1%)
**Triple negative (ductal)**	ER and PR absent (<1%)
**TNBC**	HER2 negative

List S1 – Patient tumor information

**T_ and N_stage according to AJCC 8th Edition Breast Cancer Staging System**	
**T Category**	**T Criteria**
**T1**	Tumor ≤ 20mm in greatest dimension
**T2**	Tumor > 20mm but ≤ 50mm in greatest dimension
**T3**	Tumor > 50mm in greatest dimension
**N Category**	**N Criteria**
**N0**	No regional lymph node metastasis indentified or ITCs (isolated tumor cells) only
**N1**	Micrometastases
	or metastases in 1-3 axillary lymph nodes
	and/or clinically negative internal mammary nodes with micrometastases or macrometastases by sentinel lymph node biopsy
**N2**	Metastasese in 4-9 axillary lymph nodes
	or positive ipsilateral internal mammary lymph nodes by imaging in the absence of axillary lymph node metastases
**N3**	Metastases in 10 or more axillary lymph nodes
	or in infraclavicular (Level III axillary) lymph nodes
	or positive ipsilateral internal mammary lymph nodes by imaging in the presence of one or more positive Level I, II axillary lymph nodes
	or in more than three axillary lymph nodes and micrometastases or macrometastases by sentinel lymph node biopsy in clinically negative ipsilateral internal mammary lymph nodes
	or in ipsilateral supraclavicular lymph nodes

List S2 – FACS Antibodies

Target	Fluorescence	Cat. number	Producer
CD11B	APC-CY7	557754	BD BIOSCIENCES
CD11C	PE-CY7	561356	BD BIOSCIENCES
CD14	BV785	563698	BD BIOSCIENCES
CD15	AF488	301910	BIOLEGEND
CD3	BV650	563852	BD BIOSCIENCES
CD45	PERCP-EFLUOR 710	46-0459-42	eBIOSCIENCES
CD68	PE-CF594	564944	BD BIOSCIENCES
CD8	PE-CY7	25-0088-42	eBIOSCIENCES
FIXABLE LIVE/DEAD cell marker	AMCYAN	L34957	THERMOFISHER
CD56	BV570	318330	BIOLEGEND
HLA-DR	BV650	564231	BD BIOSCIENCES
CD90	APC	559869	BD BIOSCIENCES
CD31	BV421	564089	BD BIOSCIENCES
EPCAM	BV785	324238	BIOLEGEND
CD4	PE	555347	BD BIOSCIENCES

List S3 – Drug information

	Compound name	Source	Catalog number	Mechanism of action/Target
1	Afatinib	Selleck Chemicals	S1011	EGFR/HER2 inhibitor
2	Apitolisib	Selleck Chemicals	S2696	PI3Kα/β/δ/γ and mTOR inhibitor
3	AZD8055	Selleck Chemicals	S1555	mTOR inhibitor
4	Carboplatin	Selleck Chemicals	S1215	DNA synthesis inhibitor
5	Dacomitinib	Selleck Chemicals	S2727	EGFR/HER2 inhibitor
6	Docetaxel	Selleck Chemicals	S1148	Inhibitor of depolymerisation of microtubules
7	Doxorubicin	Selleck Chemicals	S1208	DNA topoisomerase II inhibitor
8	Epirubicin	Selleck Chemicals	S1223	DNA topoisomerase inhibitor
9	Fulvestrant	Selleck Chemicals	S1191	Estrogen receptor (ER) antagonist
10	Gemcitabine	Selleck Chemicals	S1714	Nucleic acid synthesis inhibitor
11	Lapatinib	Selleck Chemicals	S2111	EGFR/HER2 inhibitor
12	Methotrexate	Selleck Chemicals	S1210	Dihydrofolate reductase (DHFR) inhibitor
13	Mitoxantrone	Selleck Chemicals	S1889	DNA topoisomerase II inhibitor
14	Navitoclax	Selleck Chemicals	S1001	Bcl-xL, Bcl-2 and Bcl-w inhibitor
15	Neratinib	Selleck Chemicals	S2150	EGFR/HER2 inhibitor
16	Omipalisib	Selleck Chemicals	S2658	p110α/β/δ/γ and mTORC1/2 inhibitor
17	Paclitaxel	Selleck Chemicals	S1150	Microtubule polymer stabilizer
18	Palbociclib	Selleck Chemicals	S1116	Highly selective inhibitor of CDK4/6
19	Pertuzumab (Perjeta)	Roche	-	HER2 inhibitor
20	Pictilisib	Selleck Chemicals	S1065	PI3Kα/δ inhibitor
21	Trastuzumab (Herceptin)	Roche	-	HER2 inhibitor
22	Venetoclax	Selleck Chemicals	S8048	Bcl-2-selective inhibitor
23	Vinorelbine	Selleck Chemicals	S4269	Mitosis inhibitor through interaction with tubulin
24	Vincristine	Selleck Chemicals	S1241	Microtubules polymerization inhibitor by binding to tubulin
25	Megestrol	Selleck Chemicals	S1304	Synthetic progestogen
26	Tamoxifen	Selleck Chemicals	S1238	Antagonist of the estrogen receptor
27	Raloxifene	Selleck Chemicals	S1227	Antagonist of the estrogen
28	Toremifene	Selleck Chemicals	S1776	Estrogen receptor modulator

29	Flutamide	Selleck Chemicals	S1908	Anti-androgen drug by binding to androgen receptor
30	Bicalutamide	Selleck Chemicals	S1190	Androgen receptor (AR) antagonist
31	Enzalutamide	Selleck Chemicals	S1250	Androgen receptor (AR) antagonist
32	Thalidomide	Selleck Chemicals	S1193	E3 ubiquitin ligase and TNF-α inhibitor
33	Capecitabine	Selleck Chemicals	S1156	Tumor-selective fluoropyrimidine carbamate
34	Fluorouracil	Selleck Chemicals	S1209	DNA/RNA synthesis inhibitor by inhibiting thymidylate synthase
35	Cyclophosphamide	Selleck Chemicals	S2057	Alkylating agent, crosslink DNA and causing strand breakage
36	Irinotecan	Selleck Chemicals	S1198	Topoisomerase 1 inhibitor
37	4-Hydroxytamoxifen	Sigma Aldrich	H7904	metabolite of tamoxifen, antiestrogen
38	Endoxifen	Sigma Aldrich	E8284	metabolite of tamoxifen, antiestrogen
39	4-hydroperoxy Cyclophosphamide	Cayman Chemical	19527	metabolite of Cyclophosphamide. Alkylating agent, crosslink DNA and causing strand breakage

References:

1. K. Cibulskis, *et al.*, Sensitive detection of somatic point mutations in impure and heterogeneous cancer samples. *Nature Biotechnology* **31**, 213–219 (2013).

2. C. T. Saunders, *et al.*, Strelka: accurate somatic small-variant calling from sequenced tumor-normal sample pairs. *Bioinformatics* **28**, 1811–1817 (2012).

3. P. Cingolani, *et al.*, A program for annotating and predicting the effects of single nucleotide polymorphisms, SnpEff: SNPs in the genome of Drosophila melanogaster strain w1118; iso-2; iso-3. *Fly* **6**, 80–92 (2012).

4. N. Sachs, *et al.*, A Living Biobank of Breast Cancer Organoids Captures Disease Heterogeneity. *Cell* **172**, 373–386.e10 (2018).

5. V. Boeva, *et al.*, Control-free calling of copy number alterations in deep-sequencing data using GC-content normalization. *Bioinformatics* **27**, 268–269 (2011).

6. S. Durinck, P. T. Spellman, E. Birney, W. Huber, Mapping identifiers for the integration of genomic datasets with the R/Bioconductor package biomaRt. *Nat. Protoc.* **4**, 1184–1191 (2009).

7. L. R. Yates, *et al.*, Subclonal diversification of primary breast cancer revealed by multiregion sequencing. *Nat. Med.* **21**, 751–759 (2015).

8. S. Nik-Zainal, *et al.*, Landscape of somatic mutations in 560 breast cancer whole-genome sequences. *Nature* **534**, 47–54 (2016).

9. S. Anders, P. T. Pyl, W. Huber, HTSeq--a Python framework to work with high-throughput sequencing data. *Bioinformatics* **31**, 166–169 (2015).

10. M. D. Robinson, A. Oshlack, A scaling normalization method for differential expression analysis of RNA-seq data. *Genome Biol.* **11**, R25 (2010).

11. M. D. Robinson, D. J. McCarthy, G. K. Smyth, edgeR: a Bioconductor package for differential expression analysis of digital gene expression data. *Bioinformatics* **26**, 139–140 (2010).

12. D. J. McCarthy, Y. Chen, G. K. Smyth, Differential expression analysis of multifactor RNA-Seq experiments with respect to biological variation. *Nucleic Acids Res.* **40**, 4288–4297 (2012).

13. A. Subramanian, *et al.*, Gene set enrichment analysis: a knowledge-based approach for interpreting genome-wide expression profiles. *Proc. Natl. Acad. Sci. U. S. A.* **102**, 15545–15550 (2005).

14. Y. Benjamini, Y. Hochberg, Controlling the False Discovery Rate: A Practical and Powerful Approach to Multiple Testing. *J. R. Stat. Soc. Series B Stat. Methodol.* **57**, 289–300 (1995).

15. E. Becht, *et al.*, Estimating the population abundance of tissue-infiltrating immune and stromal cell populations using gene expression. *Genome Biol.* **17**, 218 (2016).

16. B. Yadav, *et al.*, Quantitative scoring of differential drug sensitivity for individually optimized anticancer therapies. *Sci. Rep.* **4**, 5193 (2014).

17. A. Malyutina, *et al.*, Drug combination sensitivity scoring facilitates the discovery of

synergistic and efficacious drug combinations in cancer. *PLoS Comput. Biol.* **15**, e1006752 (2019).

18. T. Pemovska, *et al.*, Individualized systems medicine strategy to tailor treatments for patients with chemorefractory acute myeloid leukemia. *Cancer Discov.* **3**, 1416–1429 (2013).

19. A. C. Picornell, *et al.*, Breast cancer PAM50 signature: correlation and concordance between RNA-Seq and digital multiplexed gene expression technologies in a triple negative breast cancer series. *BMC Genomics* **20** (2019).

20. J. Tomfohr, J. Lu, T. B. Kepler, Pathway level analysis of gene expression using singular value decomposition. *BMC Bioinformatics* **6** (2005).
